# Phosphate accumulation in rice leaves promotes fungal pathogenicity and represses host immune responses during pathogen infection

**DOI:** 10.3389/fpls.2023.1330349

**Published:** 2024-01-17

**Authors:** Héctor Martín-Cardoso, Mireia Bundó, Beatriz Val-Torregrosa, Blanca San Segundo

**Affiliations:** ^1^ Centre for Research in Agricultural Genomics (CRAG) CSIC-IRTA-UAB-UB, C/de la Vall Moronta, CRAG Building, Campus Universitat Autònoma de Barcelona (UAB), Bellaterra (Cerdanyola del Vallés), Barcelona, Spain; ^2^ Consejo Superior de Investigaciones Científicas (CSIC), Barcelona, Spain

**Keywords:** blast, defense response, effectors, *Magnaporthe oryzae*, *Oryza sativa*, phosphate, reactive oxygen species

## Abstract

Rice is one of the most important crops in the world and a staple food for more than half of the world’s population. At present, the blast disease caused by the fungus *Magnaporthe oryzae* poses a severe threat to food security through reduction of rice yields worldwide. High phosphate fertilization has previously been shown to increase blast susceptibility. At present, however, our knowledge on the mechanisms underpinning phosphate-induced susceptibility to *M. oryzae* infection in rice is limited. In this work, we conducted live cell imaging on rice sheaths inoculated with a *M. oryzae* strain expressing two fluorescently-tagged *M. oryzae* effectors. We show that growing rice under high phosphate fertilization, and subsequent accumulation of phosphate in leaf sheaths, promotes invasive growth of *M. oryzae*. Consistent with this, stronger expression of *M. oryzae* effectors and Pathogenicity Mitogen-activated Protein Kinase (*PMK1*) occurs in leaf sheaths of rice plants grown under high a phosphate regime. Down-regulation of fungal genes encoding suppressors of plant cell death and up-regulation of plant cell death-inducing effectors also occurs in sheaths of phosphate over-accumulating rice plants. Treatment with high Pi causes alterations in the expression of fungal phosphate transporter genes potentially contributing to pathogen virulence. From the perspective of the plant, Pi accumulation in leaf sheaths prevents H_2_O_2_ accumulation early during *M. oryzae* infection which was associated to a weaker activation of *Respiratory Burst Oxidase Homologs* (*RBOHs*) genes involved in reactive oxygen species (ROS) production. Further, a weaker activation of defense-related genes occurs during infection in rice plants over-accumulating phosphate. From these results, it can be concluded that phosphate fertilization has an effect on the two interacting partners, pathogen and host. Phosphate-mediated stimulation of fungal effector genes (e.g., potentiation of fungal pathogenicity) in combination with repression of pathogen-inducible immune responses (e.g., ROS accumulation, defense gene expression) explains higher colonization by *M. oryzae* in rice tissues accumulating phosphate. Phosphate content can therefore be considered as an important factor in determining the outcome of the rice/*M. oryzae* interaction. As fertilizers and pesticides are commonly used in rice cultivation to maintain optimal yield and to prevent losses caused by pathogens, a better understanding of how phosphate impacts blast susceptibility is crucial for developing strategies to rationally optimize fertilizer and pesticide use in rice production.

## 1 Introduction

Rice is one of the most important cereal crops and a staple food for more than half of the world’s population. However, rice production is seriously threatened by rice blast caused by the fungus *Magnaporthe oryzae*, this pathogen affecting both temperate and tropical rice varieties (Wilson and Talbot, 2009). *M. oryzae* initiates its infection cycle by attaching the conidium to the host surface. Under favorable conditions, the conidium germinates and forms the germ tube which differentiates into a specialized infection structure, the appressorium, from which the penetration hypha emerges. Upon penetration, *M. oryzae* follows a hemibiotrophic lifestyle consisting of an initial biotrophic phase in which the fungus forms bulbous and branched invasive hyphae that sequentially invade living neighboring host cells. The invasive hyphae are encased by a host membrane, known as the extra-invasive hyphal membrane (EIHM) which creates an apoplastic compartment between the intracellular hyphae and the rice cell (Kankanala et al., 2007). As the bulbous intracellular invasive hyphae develops, an extended dome-shaped structure forms, known as the biotrophic interfacial complex (BIC) (Mosquera et al., 2009; Khang et al., 2010). The fungus uses plasmodesmata for cell-to-cell movement to adjacent cells (Khang et al., 2010; Fernandez and Orth, 2018; Sakulkoo et al., 2018). Then, the fungus switches to a necrotrophic lifestyle in which the fungus promotes host cell death and lesion formation (Yan and Talbot, 2016; Fernandez and Orth, 2018).

During infection, the pathogen deploys diverse strategies to facilitate infection which include the production of effectors and pathogenicity-related genes (Devanna et al., 2022). Certain fungal effectors have been shown to be important pathogenic factors by suppressing host immune responses and modulating host cell death (Deb et al., 2021; Figueroa et al., 2021; Shao et al., 2021). During biotrophic growth the fungus secretes the so-called biotrophy-associated secreted (BAS) effector proteins that are capable of manipulating plant cellular responses for its own benefit (Mentlak et al., 2012; Park et al., 2012; Fernandez and Orth, 2018; Tariqjaveed et al., 2021).

Depending on their localization *in planta*, *M. oryzae* effectors have been classified as apoplastic and cytoplasmic effectors. Apoplastic effectors are secreted into the EIHM compartment surrounding the invasive hyphae, but do not enter the plant cytoplasm (Mosquera et al., 2009; Zhang and Xu, 2014). Known apoplastic *M. oryzae* effectors are BAS4, BAS113, and Slp1 (Secreted LysM Protein 1). On the other hand, cytoplasmic effectors accumulate at the BIC structure of bulbous invasive hyphae before being translocated to the host cytoplasm of living rice cells (Mosquera et al., 2009; Khang et al., 2010). Cytoplasmic effectors include PWL2 (Pathogenicity toward Weeping Lovegrass2), Avr-Pita, BAS1 and BAS107, among others. Recent studies have shown that *M. oryzae* also secretes effectors through the BIC which are then delivered to the host cell nucleus (Lee et al., 2023). Although it is generally assumed that effectors can function in multiple ways to suppress the plant immune responses and promote infection, our understanding on factors and regulatory mechanisms influencing the *in planta* expression of fungal effector genes is still limited.

When attacked by pathogens, plants activate defense mechanisms, a process in which two different mechanisms are usually considered. The first path is induced upon recognition of conserved molecular signatures derived from microbes, known as pathogen-associated molecular patterns (PAMPs), by plasma membrane receptors in the plant cell. This recognition triggers the activation of a general defense response referred to as PAMP-triggered immunity (PTI) which provides basal immunity against pathogen infection (Jones and Dangl, 2006; Couto and Zipfel, 2016; Bentham et al., 2020). PTI activates multiple signaling pathways in the plant cell, including the accumulation of reactive oxygen species (ROS), activation of protein phosphorylation processes, and accumulation of Pathogenesis-Related (*PR*) proteins, among others (Torres et al., 2006; Van Loon et al., 2006). The second path of defense mechanisms relies on the recognition of secreted microbial effectors by intracellular receptors encoded by Resistance (*R*) genes. This recognition induces strong immune reactions, the so-called effector-triggered immunity (ETI), often associated with a hypersensitive response (HR) (Cui et al., 2015). During HR, a strong ROS burst and localized cell death occurs at infection that efficiently blocks the spreading of the pathogen, thus, leading to disease resistance.

Being a foliar pathogen, *M. oryzae* has absolute requirement for nutrients from the host tissue, so under infection conditions there is a competition for nutritional resources between the host and the pathogen. Furthermore, the fungus must develop different strategies for nutrient acquisition which are dependent on the stage of the infection process. During the initial biotrophic phase, the fungus acquires nutrients from living host cells, whereas during the necrotrophic phase, the fungus derives nutrients from dead or dying cells (Sharpee et al., 2017; Guo et al., 2019; Deb et al., 2021; Zhang et al., 2022). Regulation of fungal nutrition gene expression during infection is also documented, mainly in relation to carbon and nitrogen regulatory systems (Divon and Fluhr, 2007; Johns et al., 2021). Also, plant defense mechanisms can be exploited by fungi to ensure a ready supply of nutrients for its own use. As an example, plant defense compounds that function as ROS scavengers, such as GABA (γ-Aminobutyrate), can be taken up and metabolized by the fungus (Divon and Fluhr, 2007).

Accumulating evidence also supports that nutrient content in the plant might influence disease resistance (Val-Torregrosa et al., 2021). At present, however, it is not possible to generalize the effects of nutrients for all plant-pathogen systems, as variable effects are observed on enhancing or decreasing disease resistance in plants grown under inadequate nutrient supply. Incidentally, excess nitrogen fertilization has long been recognized to increase susceptibility to infection by *M. oryzae* in rice (Nitrogen-induced susceptibility, or NIS) through an increase of fungal pathogenicity (Ballini et al., 2013; Huang et al., 2017). More recently, high phosphate (Pi) fertilization and transgenic expression of the microRNA miR399 in rice (leading to Pi accumulation in leaves) was found to increase blast susceptibility (Campos-Soriano et al., 2020). Thus, the over-use of fertilizers in rice fields might have negative effects in rice production by facilitating blast disease, which also has a negative effect on the human health and environment. Ironically, fertilizers containing plant nutrients (e.g., nitrogen, phosphorus) are routinely used to maximize yield in rice cultivation, while pesticides are being used for the control of the rice blast disease. However, limited research has been undertaken to understand how nutrient supply, e.g., Pi supply, might affect blast severity. For instance, no information is currently available on whether Pi fertilization has an effect on the production of *M. oryzae* effectors and what the impact of this regulation could be on blast resistance. A better knowledge on the underlying mechanisms is of great significance for sustainable agricultural practices less dependent on pesticides and fertilizers.

In this study we investigated whether Pi accumulation in rice leaf sheaths modulates the expression of *M. oryzae* effectors and Pi transporters during infection. Using live-cell imaging of rice sheaths infected with a *M. oryzae* isolate expressing fluorescent protein-tagged effectors (e.g., BAS4 and PWL2), we demonstrated that treatment of rice plants with high Pi, and subsequent accumulation of Pi in rice tissues, promotes host cell invasion. Of interest, Pi accumulation in host tissues fosters the expression of *M. oryzae* effectors and oppositely modulates the expression of suppressors of plant cell death and plant cell-death-inducing effectors (down-regulation and up-regulation, respectively). In addition to fungal pathogenicity, Pi treatment influences the expression of fungal Pi transporters. Stronger expression of Pi transporters in the Major Facilitator Superfamily (MFS) occurs in rice plants that have been grown under Pi limiting conditions. We also show that Pi accumulation in rice leaf sheaths negatively affects the capability of the rice plant to activate defense gene expression during pathogen infection. At the cytological level, Pi accumulation in leaf sheaths prevents ROS accumulation early during biotrophy. Together, results here presented support that treatment of rice plants with high Pi has an effect on the two interacting partners, host and pathogen. These findings provide new insights to understand the negative effect that high Pi fertilization has on blast incidence which, in turn, would affect rice production.

## 2 Materials and methods

### 2.1 Plant and fungal material

Rice (*Oryza sativa*) was grown at 28°C/25°C with a 14 h/10 h light/dark cycle, and 60% humidity. The *japonica* cultivars Maratelli, Nipponbare and Tainung 67 (TN67) were used in this work. The rice blast fungus *M. oryzae* (strain Guy 11) was routinely grown on Complete Medium Agar (CMA, containing 30 mg/L chloramphenicol) for 15 days at 25°C under a 16 h/8 h light/dark cycle. *M. oryzae* spores were collected from fungal mycelium by adding sterile water to the mycelium surface, filtered with Miracloth and adjusted to the desired concentration using a Neubauer counting chamber. For infection assays, Tween® 20 was added to the spore suspension at a final concentration of 0.02%.

### 2.2 Transformation of *M. oryzae*

The plasmid pBV591 (kindly provided by Dr. B. Valent) was used for expression of fluorescently-labelled *M. oryzae* effectors. This plasmid contains the *MoPWL2* gene fused to mCherry and the *MoBAS4* gene fused to eGFP. Both genes are expressed under the control of their own promoter (Khang et al., 2010). The pBV591 plasmid harbors the hygromycin gene as the selectable marker. Transformation of *M. oryzae* was carried out using *Agrobacterium tumefaciens* (strain AGL-1) following the protocol previously described (Campos-Soriano and San Segundo, 2009) with minor modifications. Briefly, the *M. oryzae* spore suspension (500 μL, 10^6^ spores/mL) was mixed with 500 μL of *A. tumefaciens* suspension and spread onto a sterile filter paper (co-cultivation medium plate). After 2 days of co-cultivation at 24°C, the filter paper was transferred to a selection medium plate (Potato Dextrose Agar, PDA), containing hygromycin B (at a final concentration of 250 μg/mL). Hygromycin-resistant colonies were then individually grown on 24-well plates in selection medium containing hygromycin B.

### 2.3 Pi treatment and measurement of Pi content

**Figure 1:**
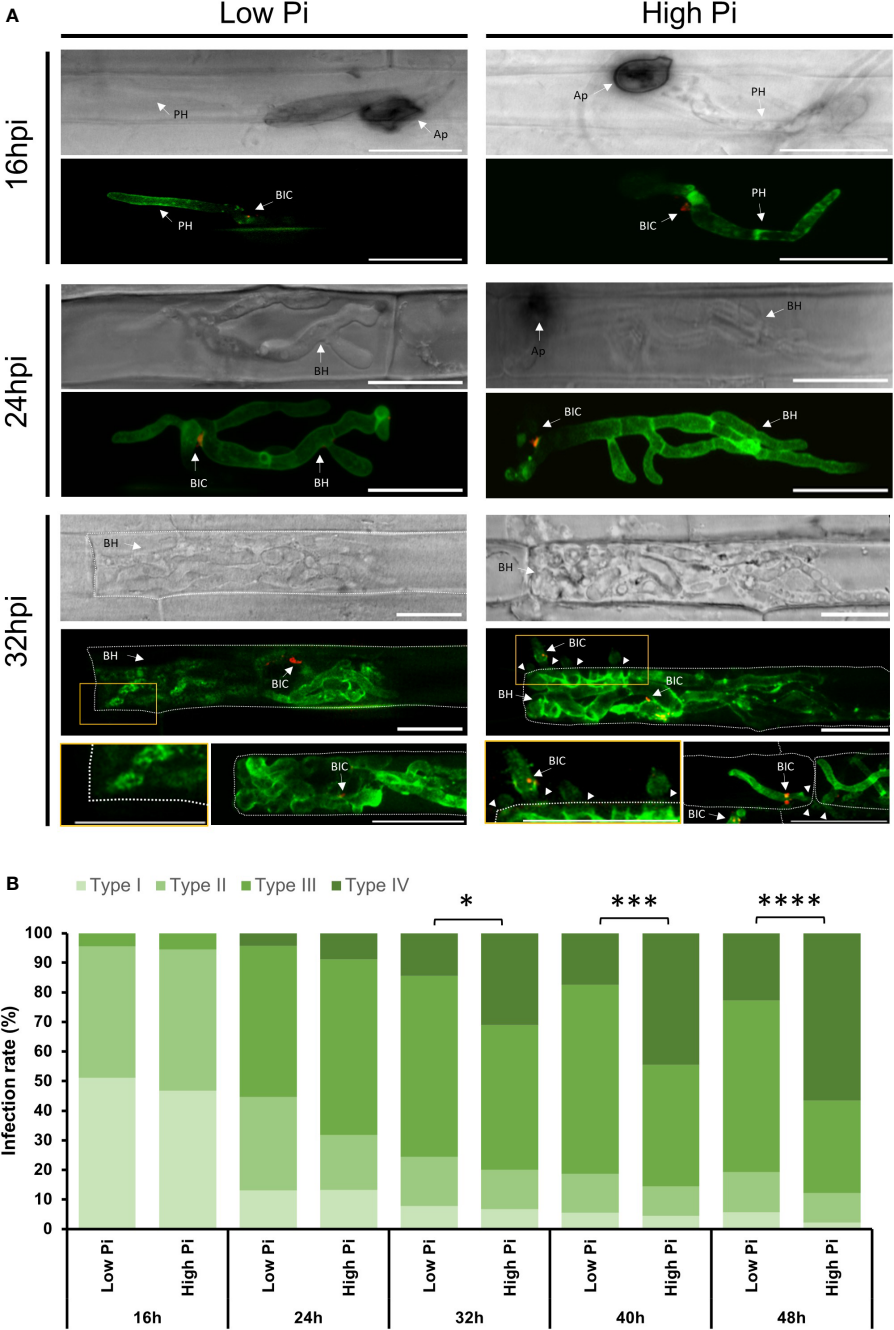
Life cell imaging of *M. oryzae*-inoculated rice sheaths from Low-Pi and High-Pi plants. Plants were watered for 7 days and then fertilized with modified Hoagland half-strength solution supplemented with 0.025 mM Pi (for low-Pi treatment) and 2.5 mM Pi (for high-Pi treatment) for 14 days. Excised rice sheaths were inoculated with spores of a compatible *M. oryzae* strain (Guy11; 5 x 10^4^ spores/ml) expressing *BAS4-GFP* and *PWL2-mCherry* under the control of its own promoter. Confocal microscopy was used to monitor biotrophic invasion of rice cells (16, 24 and 32 hpi). **(A)** Life cell imaging of *M. oryzae*-inoculated rice sheaths. GFP fluorescence from the apoplastic effector BAS4 accumulating at the EIHM compartment outlined invasive hyphae, whereas the cytoplasmic effector PWL2 accumulated at BICs (red fluorescence). Images correspond to infection experiments on leaf sheaths of the rice cultivar Maratelli. Same results were obtained on leaf sheaths of the rice cultivars Nipponbare and Tainung 67 (TN67). Scale bars represent 20 µm. Ap, Appressorium; BIC, Biotrophic Interfacial Complex; BH, Bulbous Hyphae; PH, Primary Hyphae. Arrowheads indicate cell to cell of *M. oryzae*. **(B)** Infection events were classified into 4 types: Type I, Appressorium formation; Type II, Successful penetration with primary invasive hyphae formed in the first invaded host cell; Type III: Formation of bulbous, branched hyphae; and Type IV, hyphae extending to neighboring cells. Frequencies are expressed as percentages of total infection sites (penetrated cells). Three independent experiments were carried out with similar results. Asterisks indicate significant differences as determined by χ2 test (n = 100 each experiment; *, P < 0.05; ***, P < 0.001; ****, P < 0.0001).

For Pi treatment, plants were grown for 21 days in a mixture of 50% turface and vermiculite (>50 mg/L phosphorus) and 50% quartz sand and watered for 7 days. The plants were then fertilizer for 15 days with a Hoagland half-strength solution (2.5 mM KNO_3_, 2.5 mM Ca(NO_3_)_2_·4H_2_O, 1 mM MgSO_4_·7H_2_O, 0.5 mM NH_4_NO_3_, 23.15 μM H_3_BO_3_, 4.55 μM MnCl_2_·4H_2_O, 0.38 μM ZnSO_4_·7H_2_O, 0.1 μM CuSO_4_·5H_2_O, 0.14 μM Na_2_MoO_4_·2H_2_O, 26 μM Fe-EDDHA, pH 5.5) containing the desired Pi concentration, namely 0.025 mM Pi for low-Pi, and 2.5 mM Pi for high-Pi (the final concentration of Pi was adjusted using KH_2_PO_4_) (**Suppplementary Figure 1**).Free Pi content in rice leaf sheaths was determined as previously described (Ames, 1966; Versaw and Harrison, 2002). Briefly, the rice leaf sheaths were grounded in liquid nitrogen and the leaf power (50 mg) was treated with 1% glacial acetic acid. An ammonium molybdate/ascorbic acid solution (0.7 mL) (Versaw and Harrison, 2002) was added to the solution and the absorbance at 820 nm was measured.

For measurement of Pi content in *M. oryzae* mycelia, the fungus was grown for 15 days in Complete Media (CM) containing different Pi concentrations (0.1 mM Pi, 0.5 mM Pi, 0.75 mM Pi and 1 mM Pi). In control cultures (0 mM Pi), Pi was not added to the culture medium. Pi content quantification in fungal mycelia was performed as for rice tissues. Statistical significances were calculated by Student’s *t*-test.

### 2.4 Infection assays and live-cell imaging of *M. oryzae* effectors *in planta*

Leaf sheaths from rice plants (Maratelli, Nipponbare and TN67 cultivars) at the 3-4 leaf stage were inoculated with *M. oryzae* spores (150 µl, 5 x 10^4^ spores/mL) as previously described (Kankanala et al., 2007). Confocal imaging was performed with a Leica SP5 confocal microscope using excitation/emission wavelengths of 488 nm/500 to 535 nm for GFP, and 561 nm/575 to 646 nm for mCherry. Images were analyzed using the ImageJ software (National Institute of Health, Bethesda, MD, USA; https://imagej.nih.gov/ij/). Three independent experiments were performed with at least 5 biological replicates, each one from a pool of at least three leaf sheaths. Events during biotrophic invasion of leaf sheaths (Type I to Type IV) were examined on at least 100 infection sites/sample. The Chi-squared (χ2) test was used to assess statistical significance.

For determination of fungal biomass, genomic DNA was extracted from *M. oryzae*-infected leaf sheaths as described by Murray and Thompson (1980) but using MATAB as the extraction buffer (0.1 M Tris-HCl pH 8.0, 1.4 M NaCl, 20 mM EDTA, 2% MATAB, 1% PEG 6000 and 0.5% Na_2_SO_3_). Fungal biomass was quantified by quantitative PCR (qPCR) using specific primers for the *M. oryzae* 28S DNA gene (Qi and Yang, 2002). Primers used for qPCR are listed in **Supplementary Table 1**. Statistical analysis was performed using Student’s *t*-test.

### 2.5 Expression analysis by RT-qPCR

Total RNA was extracted using the Maxwell RSC Plant RNA Kit (Promega). Total RNA (1 μg) was retrotranscribed using the High Capacity cDNA reverse transcription Kit (Applied Biosystems, Waltham, MA, USA). Primers were designed using Primer-BLAST (https://www.ncbi.nlm.nih.gov/tools/primer-blast/). RT-qPCR was performed in optical 96-well plates using SYBR® green in a LightCycler 480 (Roche, Basel, Switzerland). The *ubiquitin1* gene (*OsUbi1*, Os06g0681400) and the *M. oryzae actin* gene (*MoActin*, MGG_03982) were used to normalize transcript levels for expression analysis of rice and *M. oryzae* genes, respectively. Four biological replicates each one from a pool of three different plants, and three technical replicates for each biological replicate were analyzed. Primers used for RT-qPCR are listed in **Supplementary Table 1**. Two-way ANOVA followed by Tukey’s honestly significant difference (HSD) and Student’s *t*-test were used to assess statistical significance of RT-qPCR data where appropriate.

### 2.6 Histochemical analysis of ROS accumulation

Cellular ROS (H_2_O_2_) accumulation was visualized using 3,3′-diaminobenzidine (DAB) staining method (Wang et al., 2019) with the following modifications. Rice leaf sheaths (3 cm segments) were rinsed with sterile water (three times) and incubated in DAB solution (1 mg/mL; pH 3.8) for 24 h. Samples were washed with 100% ethanol (approx. 24 h, until the tissue becomes transparent) and stored in 50% glycerol until visualization. For quantification of dead cells in *M. oryzae*-infected leaf sheaths, the number of dead cells with cellular aggregates (dark brown color) was determined in DAB-stained leaf sheaths (Dangol et al., 2019; Wang et al., 2019; Chen et al., 2022). For this, a total of 15 visual fields were observed for each leaf sheath using a Leica DM6 microscope under bright field illumination. Four biological replicates, each one from a pool of three leaf sheaths were used. Significant differences were evaluated with Student’s *t*-test.

### 2.7 Lipid peroxidation assay

The level of lipid peroxidation was determined by measuring the amount of MDA produced using the thiobarbituric acid reagent (Campo et al., 2014). Briefly, samples (0.05 g) were homogenized in 1 mL of 80% (v/v) ethanol and centrifuged at 16,000 g for 20 min at 4°C. The supernatant (0.5 mL) was mixed with 0.5 mL of 20% (w/v) trichloroacetic acid containing 0.65% (w/v) thiobarbituric acid. The mixture was incubated at 85°C for 30 min and quickly cooled in an ice bath. After centrifugation (10,000 g, 10 min), the absorbance of the supernatant was measured at 532 nm and 600 nm (Spectramax M3 reader, Molecular Devices, USA). Nonspecific absorption at 600 nm was subtracted from the 532 nm readings. The MDA content was calculated using its molar extinction coefficient (156 mM^−1^ cm^−1^). Results are expressed as mM MDA/g fresh weight (FW). Two-way ANOVA followed by Tukey’s HSD test was used to analyze data.

### 2.8 *In vitro* growth of *M. oryzae* and calcofluor white staining

To examine the effect of Pi on the *in vitro* growth of *M. oryzae* we used a microtiter well plate assay. For this, the fungus was grown on CMA as described above and spores were collected by adding sterile water to the surface of the mycelium. In microtiter wells, fungal cultures consisted of Complete Media (CM) and *M. oryzae* spores at a final concentration of 5 x 10^4^ spores/mL (in a total volume of 200 µL), with different Pi (KH_2_PO_4_) concentrations: 0 mM Pi (no Pi added), 0.1 mM Pi, 0.5 mM Pi, 0.75 mM Pi and 1 mM Pi. *M. oryzae* was grown for 48 h at 25°C under dark conditions, and the absorbance was measured at 595 nm at different timepoints (Spectramax M3 reader, Molecular Devices, USA). Negative controls consisted of media at each Pi concentration without *M. oryzae* spores. Five experiments were performed, each one with 12 technical replicates. Data were analyzed using Student’s *t*-test.

To assess the effect of Pi on spore germination, fungal spores were germinated on Complete Medium (CM) for 2 h, 4 h and 6 h with no Pi added, or with Pi added to a final concentration of 1 mM Pi. Spore germination was examined on a Leica DM6 microscope under bright field illumination. Four experiments were performed, each one with three technical replicates (N = 100 spores each replicate). Significant differences were determined using Student’s *t*-test.

Hyphal length and branching of fungal cultures were examined by Calcofluor White staining (at a final concentration of 10 µg/mL) using a Leica DM6 microscope under bright field an UV light illumination. Excitation/emission wavelengths were 340 to 380 nm/425 nm for UV light. Image analysis was performed using ImageJ software (National Institute of Health, Bethesda, MD, USA; https://imagej.nih.gov/ij/). Four assays were performed, each one with 3 technical replicates (N = 100 spores each replicate). Statistical analysis was performed using Student’s *t*-test.

To record area of mycelial growth, *M. oryzae* was grown on square Petri dishes containing Complete Media Agar (CMA) with different Pi concentrations, namely 0 mM (no Pi added), 0.1 mM, 0.5 mM, 0.75 mM and 1.0 mM Pi. *M. oryzae* was grown for 5 days at 25°C under dark conditions. Three independent experiments were performed, each one with five technical replicates. Statistical analyses were performed using One-way ANOVA.

### 2.9 Identification of Pi transporters in *M. oryzae*

Genes encoding *M. oryzae* Pi transporters were retrieved from the Joint Genome Institute (https://mycocosm.jgi.doe.gov/Magor1/Magor1.home.html; *M. oryzae* strain 70-15 genome, v3.0). According to the Transporter Classification Database (TCDB) in the JGI, Pi transporters group into five Families with the following KOG (Eukaryotic Orthologous Group) annotations: The Major Facilitator Subfamily (MFS; Group 2.A.1), The Inorganic Phosphate Transporter (PiT) Family (Group 2.A.20), The mitochondrial Carrer (MC) Family (Group 2.A.29), The Divalent Anion : Na^+^ Symporter (DASS) Family (Group 2.A.47) and The Phosphate Permease (Pho1) Family (Group 2.A.94) (**Supplementary Table 2**). Protein sequences of *M. oryzae* Pi transporters were obtained from NCBI, and further analyzed using the InterPro databases and tools provided by the member databases: InterPro, CATH-Gene3D, PANTHER and PROSITE. Gene Ontology (GO) Terms were also obtained from InterPro databases.

## 3 Results

### 3.1 Treatment of rice plants with high Pi fosters invasive growth of *M. oryzae*

To determine whether treatment of rice plants with high Pi has an effect on fungal pathogenicity, we first monitored the production of *M. oryzae* effectors during biotrophic invasion of rice cells. For this, we used life-cell imaging of rice leaf sheaths inoculated with a *M. oryzae* isolate that express effector:fluorescent protein genes, a strategy that has proven to be an outstanding tool to investigate the dynamics of effector production during *M. oryzae* infection (Mosquera et al., 2009; Khang et al., 2010; Giraldo et al., 2013).

In this work, we generated a *M. oryzae* isolate (Guy 11 strain) expressing two fluorescently labelled *M. oryzae* effectors: the BAS4 effector fused to GFP and the PWL2 effector fused to mCherry. The BAS4 and PWL2 effectors are widely used as marker genes of biotrophic growth of *M. oryzae*. Whereas the BAS4 effector accumulates at the EIHM (apoplastic effector), the PWL2 effector accumulates at the BICs (cytoplasmic effector) (Mosquera et al., 2009; Khang et al., 2010; Giraldo et al., 2013). As effector genes are expressed at specific stages during the infection process, each fluorescently-tagged effector was expressed under the control of its own promoter. Components of the expression plasmid harboring the GFP-tagged BAS4 (*BAS4:GFP*) and the mCherry-tagged PWL2 (*PWL2:mCherry*) genes have been described elsewhere (Mosquera et al., 2009; Khang et al., 2010). The expression construct was introduced into *M. oryzae* by *Agrobacterium*-mediated transformation as previously described (Campos-Soriano and San Segundo, 2009). The *PWL2:mCherry*-*BAS4:GFP-*expressing *M. oryzae* isolate was then used to monitor fungal development and invasion in cells of rice leaf sheaths. For this, the rice (*O. sativa*) plants were grown in soil for 21 days. During the last 14 days, the plants were supplied with either low Pi (0.025 mM Pi) or high Pi (2.5 mM Pi) (hereinafter referred to as Low-Pi and High-Pi plants) (**Supplementary Figure 1A**). Measurement of Pi content confirmed Pi accumulation in leaf sheaths of High-Pi compared with Low-Pi plants (**Supplementary Figure 1B**). Excised leaf sheaths of High-Pi or Low-Pi plants were then inoculated with the fluorescently-labelled *M. oryzae* isolate, and invasion of leaf sheath cells was followed with time by confocal microscopy.

Analysis of *M. oryzae*-inoculated leaf sheaths revealed filamentous primary invasive hypha in the first invaded host cell showing BAS4:GFP fluorescence, supporting that BAS4:GFP was indeed produced and secreted into the EIHM (**Figure 1A**, 16 hpi). Filamentous primary invasive hypha differentiated into bulbous and branched invasive hyphae outlined by BAS : GFP fluorescence. Consistent with the fact that PWL2 is a cytosolic effector, invasive hyphae showed accumulation of PWL2:mCherry at the BICs (**Figure 1A**). By 24 hpi, there were no important differences in the development of invasive hyphae between High-Pi and Low-Pi conditions. Only stronger GFP fluorescence could be observed in hyphae colonizing host cells in High-Pi plants compared with hyphae colonizing Low-Pi plants (**Figure 1A**, 24 hpi). However, at a later stage of infection, the movement of *M. oryzae* hyphae to adjacent cells was frequently observed in High-Pi plants, but not in Low-Pi plants (**Figure 1A**, 32 hpi; **Supplementary Figure 2**).

To further examine biotrophic invasion of rice leaf sheaths by the blast fungus, we classified the process into 4 four events: Type I, Appressorium formation; Type II: Successful penetration with primary invasive hyphae in the first invaded host cell; Type III: Formation of bulbous, branched invasive hyphae; and Type IV, hyphae invading adjacent cells. Appressoria formation in High-Pi plants did not differ from that in Low-Pi plants (**Figure 1B**, Type I). Approximately 100 appressoria/cm of leaf sheath were detected in sheaths of Low-Pi and High-Pi rice plants (**Supplementary Figure 3**), indicating that Pi treatment of rice plants likely has no effect on appressoria formation. At 16 hpi, the proportion of penetrated cells showing primary filamentous invasive hyphae was similar in High-Pi and Low-Pi plants (**Figure 1B**, Type II). At 24 hpi, however, the proportion of penetrated cells showing bulbous branched hyphae was higher in High-Pi plants than in Low-Pi plants (59,39% and 51,11%, respectively) (**Figure 1B**, Type III). Differences in the invasion of contiguous cells by fungal hyphae between High-Pi and Low-Pi were more evident at subsequent time points of the infection process (e.g., from 32 to 48 hpi) (**Figure 1B**, Type IV). At 48 hpi, 56,67% of invasive hyphae already penetrated the neighboring cells in High-Pi plants, whereas only 22,76% of the invasive hyphae expanded into contiguous cells in Low-Pi plants (**Figure 1B**, Type IV). These observations were indicative of higher rates of hyphal movement into adjacent cells in High-Pi plants compared to Low-Pi plants.

**Figure 2:**
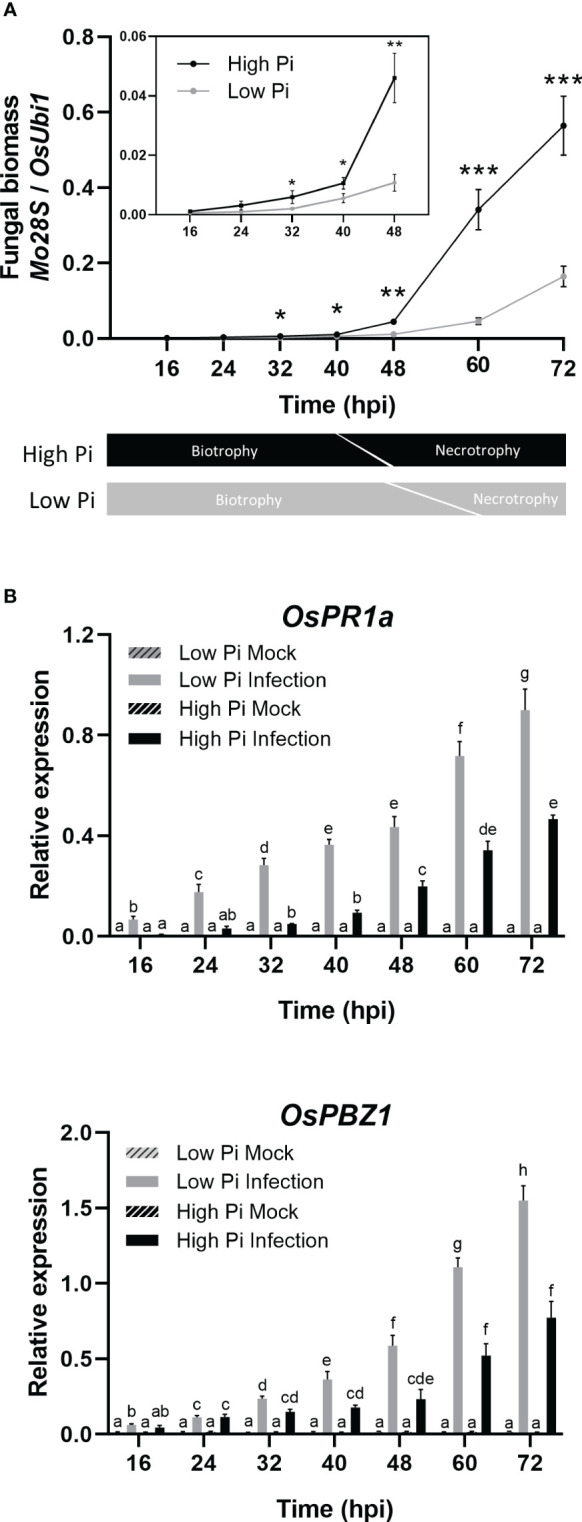
Quantification of fungal biomass and expression of rice defense genes during infection of rice (*O. sativa* cv Maratelli) leaf sheaths by *M. oryzae*. Same results were obtained on leaf sheaths of the rice cultivars Nipponbare and Tainung 67 (TN67). **(A)** Quantification of fungal DNA was performed by qPCR (upper panel) using specific primers or the *M. oryzae Mo28S* DNA gene and normalized to rice *ubiquitin 1* gene (Qi and Yang, 2002). Lower panel: schematic representation of the timing in the transition from biotrophy to necrotrophy in *M. oryzae* in High-Pi and Low-Pi rice plants. Three independent experiments were carried out with similar results. Bars represent mean ± SEM of 8 biological replicates per Pi treatment (Student’s *t*-test; *, P < 0.05; **, P < 0.01; ***, P < 0.001). **(B)** *OsPR1a* and *OsPBZ1* expression in leaf sheaths of mock‐inoculated and *M. oryzae*‐inoculated plants. Bars represent mean of 4 biological replicates (each one from a pool of three different plants) ± SEM (Two-way ANOVA followed by Tukey’s HSD test). Letters indicate significant differences among conditions.

Quantification of fungal biomass confirmed higher fungal biomass in leaf sheaths from High-Pi plants compared to Low-Pi plants which would be consistent with higher tissue colonization in High-Pi plants (**Figure 2A**, upper panel). This analysis also revealed a sudden increase in measurable fungal biomass during the infection process, but the onset of this increase differed between High-Pi plants (by 40 hpi - 48 hpi) and Low-Pi plants (by 60 hpi - 72 hpi). In other studies, it has been reported that the transition from biotrophy to necrotrophy of hemibiotrophic pathogens directly correlates with a sudden increase in fungal biomass (Yang et al., 2013; Chowdhury et al., 2017). For instance, in sesame varieties infected with *Macrophomina phaseolina*, fungal biomass increased earlier in susceptible cultivars than in resistant cultivars, which correlated well with an advancement in transition from biotrophy to necrotrophy during infection in susceptible cultivars (Chowdhury et al., 2017). On this basis, earlier increase in fungal biomass in *M. oryzae*-infected leaf sheaths might be indicative of an advancement in the transition from biotrophy to necrotrophy in High-Pi plants (**Figure 2A**, lower panel). Then, *M. oryzae* would adapt its strategy of infection according to Pi levels in the host tissue by advancing the biotrophy-necrotrophy switch in rice leaf sheaths accumulating Pi.

In an attempt to understand whether differences in host tissue colonization by *M. oryzae* between High-Pi and Low-Pi plants could be attributed to a different response in the host plant, we investigated the expression of defense-related rice genes in the fungal-infected leaf sheaths, namely *OsPR1a* and *OsPBZ1*. As previously mentioned, the induction of *PR* expression is an ubiquitous response to pathogen infection in plants, the *OsPR1* and *OsPBZ1* genes being considered markers of the induction of rice defense against *M. oryzae*. As shown in **Figure 2B**, a weaker activation of *OsPR1a* and *OsPBZ1* occurs in *M. oryzae*-infected leaf sheaths of High-Pi plants compared with *M. oryzae*-infected leaf sheaths of Low-Pi plants. Repression of fungal-inducibility of PR genes in High-Pi plants would facilitate colonization of leaf sheaths by *M. oryzae*.

From these results, it can be concluded that treatment with high-Pi, and subsequent increase in Pi content in leaf sheaths, promotes higher rates of host tissue invasion by *M. oryzae* and causes an advancement in the lifestyle transition in the pathogen. Increasing Pi content in leaf sheaths also reduces the ability of the host plant to defend itself against *M. oryzae*.

### 3.2 Phosphate accumulation in rice leaf sheaths modulates the expression of *M. oryzae* effector genes

**Figure 3:**
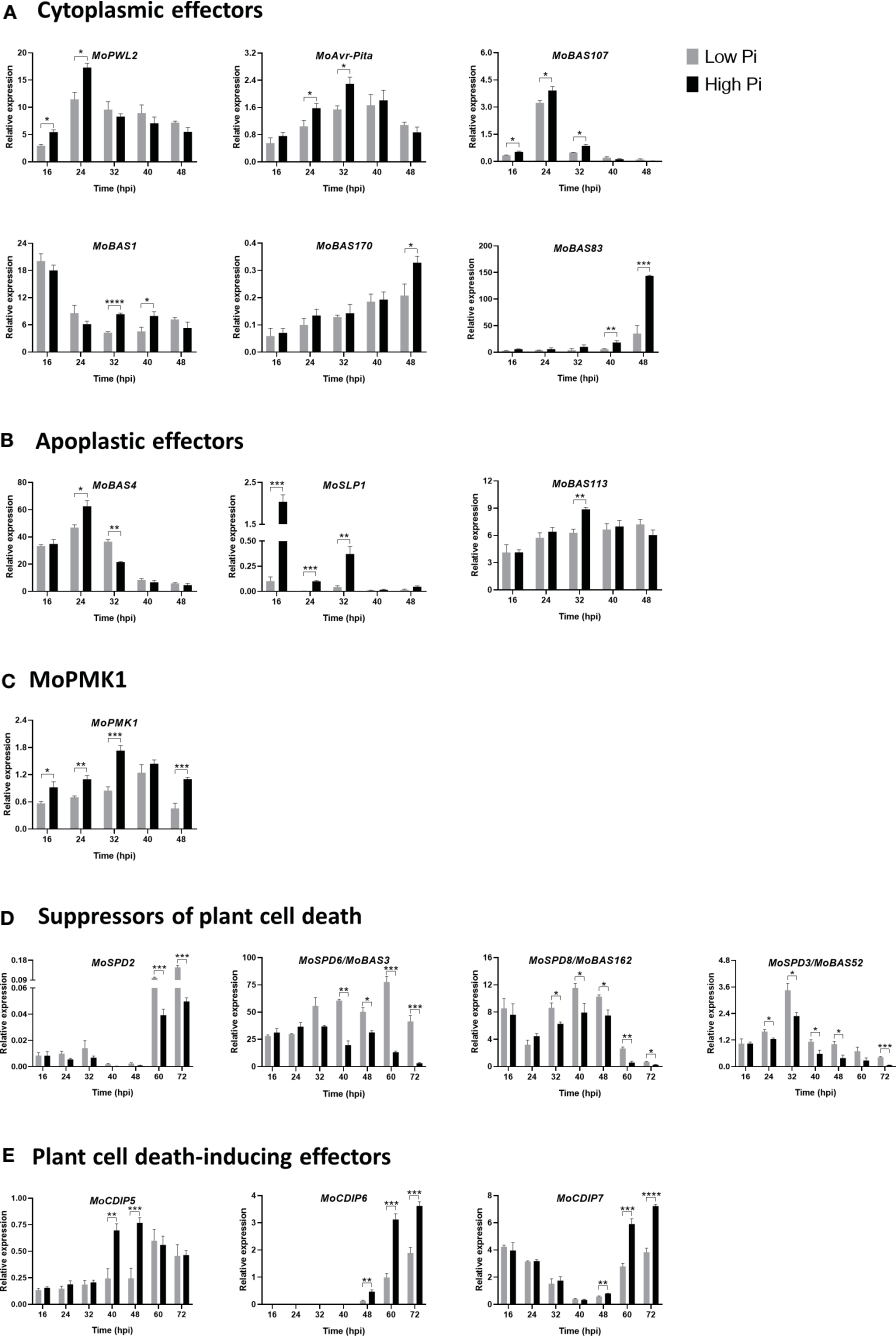
Expression of *M. oryzae* stage-specific effector genes in infected leaf sheaths at different times post-inoculation with *M. oryzae* spores (5 x 10^4^ spores/mL). Results correspond to infection experiments on leaf sheaths of the rice cultivar Maratelli. Same results were obtained on leaf sheaths of the rice cultivars Nipponbare and Tainung 67 (TN67). RT-qPCR was carried out using the *M. oryzae* actin gene (MGG_03982) as the internal control. **(A)** Cytoplasmic effectors: *MoPWL2*, *MoAvr-Pita, MoBAS1, MoBAS107*, *MoBAS170* and *MoBAS83*. **(B)** Apoplastic effectors: *MoBAS4*, *MoSLP1* and *MoBAS113.* **(C)** *MoPMK1* expression. **(D)** Suppressors of plant cell death: *MoSPD2, MoSPD6/MoBAS3*, *MoSPD8/MoBAS162* and *MoSPD3/MoBas52* (also known as *MoHEG13*). **(E)** Plant cell death-inducing effectors: *MoCDIP5*, *MoCDIP6* and *MoCDIP7.* Bars represent mean ± SEM of 4 biological replicates, each one from a pool of three different plants (Student’s *t*-test; *, P < 0.05; **, P < 0.01; ***, P < 0.001; ****, P < 0.0001).

In this work, we investigated whether Pi content in rice leaf sheaths influences the expression of *M. oryzae* effector genes. Initially, we examined the expression of *M. oryzae* effectors that are known to function in the biotrophic stage of rice infection, both cytoplasmic and apoplastic effectors. Cytoplasmic effectors examined in this study were: *MoPWL2*, *MoAvr-Pita*, *MoBAS107, MoBAS1, MoBAS170* and *MoBAS83*. Early during biotrophy, stronger expression of *MoPWL2*, *MoAvr-Pita*, and *MoBAS107* could be observed in High-Pi plants than in Low-Pi plants (**Figure 3A**; 16 - 32 hpi). Afterwards, their expression progressively decreased, and reached similar levels of expression in the two Pi conditions (**Figure 3A**). Equally, *MoBAS1*, *MoBAS170* and *MoBAS83* were expressed at a higher level in High-Pi plants at one or another time of biotrophic invasion (e.g., *MoBAS1*, at 32 - 40 hpi; *MoBAS170* at 48 hpi; and *MoBAS83* at 40 - 48 hpi) (**Figure 3A**).

Apoplastic effectors investigated in this work were: *MoBAS4*, *MoSLP1* (*Secreted LysM Protein 1*) and *MoBAS113.* Stronger expression of *MoBAS4* occurred in leaf sheaths of High-Pi plants, and then its expression decreased (**Figure 3B**). A higher expression of *MoBAS4* at 24 hpi in High-Pi plants correlates well with the observed increase in BAS : GFP fluorescence in High-Pi plants compared to Low-Pi plants. It is unlikely, however, that the effect of plant Pi content on *MoBAS4* expression might influence the quantification of infection events observed in High-Pi compared to Low-Pi plants (shown in **Figure 1B**). Regarding SLP1, this effector is known to bind chitin released from the fungal hyphae, thus, competing with chitin recognition by the receptor protein in the rice cell and leading to suppression of chitin-induced PTI (Mentlak et al., 2012; Giraldo et al., 2013). We noticed that, early during biotrophy, *MoSLP1* consistently exhibited a significantly higher expression in High-Pi plants compared to that in Low-Pi plants (**Figure 3B**; 16 – 32 hpi). A priori, a higher expression of *MoSLP1* in High-Pi plants would result in stronger suppression of chitin-induced host defense responses which, in turn, would facilitate biotrophic invasion of leaf sheaths by *M. oryzae*. Finally, the expression of *MoBAS113* was also higher in High-Pi plants than in Low-Pi plants (**Figure 3B**; 32 hpi).

From these results, it appears that the expression of *M. oryzae* effector genes is tightly regulated during biotrophy, and that Pi accumulation in the host tissue has an stimulatory effect on the expression of fungal effector genes. Presumably, Pi-mediated alterations in the expression pattern of effector genes might allow these effectors to exert their function specifically at each stage of the infection process also depending on the Pi status in the host tissue.

On the other hand, the fungal MAP Kinase *MoPMK1* (*M. oryzae Pathogenicity MAP kinase 1*) has been shown to control cell-to-cell movement *via* plasmodesmata of *M. oryzae* invasive hyphae and to regulate the expression of fungal effectors that are able to suppress rice immune responses (Sakulkoo et al., 2018; Eseola et al., 2021; Osés-Ruiz et al., 2021). As shown in **Figure 3C**, Pi accumulation in the host tissue promotes *MoPMK1* expression.

Collectively, results here presented indicated an overall effect of Pi treatment in rice plants by stimulating the expression of *M. oryzae* effectors, both cytoplasmic and apoplastic effectors, as well as *MoPMK1*. A higher expression of fungal effectors in High Pi conditions would enhance *M. oryzae* virulence and promote infection. This would be consistent with the phenotype of blast susceptibility that occurs in rice plants that have been grown under a high Pi fertilization regime (Campos-Soriano et al., 2020).

### 3.3 Phosphate content in rice leaf sheaths alters the expression of suppressors of plant cell death and cell death-inducing effector genes

The control of host cell death is of paramount importance for successful colonization of host tissues by hemibiotrophic pathogens. As part of its infection strategy, the fungus must be capable of suppressing host cell death during biotrophic invasion, while inducing plant cell death during the necrotrophic phase of infection (Fernandez and Orth, 2018; Zhang et al., 2022; Yan et al., 2023). Along with this, *M. oryzae* has been shown to produce suppressors of plant cell death (SPD) and cell death-inducing protein (CDIP) effectors whose expression needs to be tightly controlled at each step of the infection process (Sharpee et al., 2017; Guo et al., 2019; Deb et al., 2021).

In this work, we investigated whether Pi content in rice tissues has an effect on the expression of genes encoding suppressors of plant cell death (SPD genes). Expression analysis revealed distinct expression profiles of *MoSPD2*, *MoSPD6/MoBAS3*, *MoSPD8/MoBAS162*, and *MoSPD3/MoBAS52* (also named Hypothetical Effector Gen 13, *MoHEG13*) during *M. oryzae* infection (from 16 hpi to 72 hpi) (**Figure 3D**). In particular, *MoSPD2* was expressed mainly at late time points of infection (e.g., 60 to 72 hpi), while *MoSPD3/MoBAS52* was more expressed at the early time points (e.g., 24 to 32 hpi). Most importantly, a generalized down-regulation occurs in the expression of all four suppressors of plant cell death genes in High-Pi plants compared with Low-Pi plants.

Next, we monitored expression of plant cell death-inducing protein (CDIP) effectors in leaf sheaths of High-Pi and Low-Pi rice plants. They were: *MoCDIP5*, *MoCDIP6* and *MoCDIP7* genes (Chen et al., 2013; Guo et al., 2019). Contrary to what was observed on suppressors of plant cell death genes, treatment of rice plants with high Pi is associated with stronger induction of *CDIP* effector genes, a response that is more evident at the late biotrophic-early necrotrophic stages of fungal infection (*MoCDIP5*, 40 – 48 hpi; *MoCDIP6* and *MoCDIP7*, 48 – 72 hpi) (**Figure 3E**).

Collectively, this study demonstrated that Pi accumulation on leaf sheaths has an impact on the expression of suppressors of plant cell death and plant cell death-inducing effectors, and that their expression is oppositely regulated by Pi during infection (down-regulation and up-regulation, respectively). Reduced expression of suppressors of cell death during biotrophy and increased expression of cell death-inducing effectors during necrotrophy in High-Pi plants is expected to promote host tissue colonization by *M. oryzae*.

### 3.4 Cytological response to *M. oryzae* infection in leaf sheaths of High-Pi rice plants

In addition to examining the effect of Pi on host cell invasion and expression of *M. oryzae* pathogenicity genes, we also investigated whether Pi accumulation has an effect on cytological responses to *M. oryzae* infection, focusing on ROS accumulation. ROS production is a typical defense response that plants use to prevent invasion and spread of pathogens, also linked to membrane lipid peroxidation. Among ROS, H_2_O_2_ serves as an antimicrobial agent, inducer of cross-linking of cell wall components, and signaling molecule for the activation of plant defense responses. Additionally, ROS accumulation at the infection site triggers localized cell death and restricts fungal growth, this reaction being particularly useful against biotrophs (Torres et al., 2002; Vargas et al., 2012).

**Figure 4:**
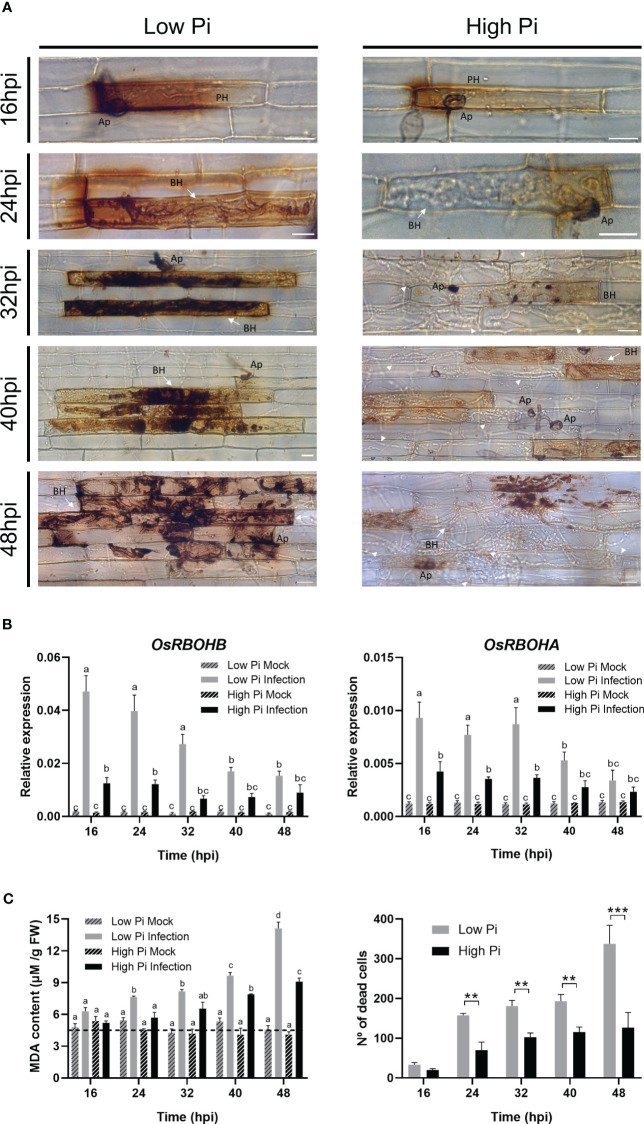
ROS accumulation, expression of *OsRBOH* genes, MDA accumulation and cell death in leaf sheaths of Low-Pi and High-Pi rice (*O. sativa* cv Maratelli) plants after inoculation with *M. oryzae* spores (5 x 10^4^ spores/ml). Statistically significant differences in **(B, C)** were determined by two-way ANOVA followed by Tukey’s HSD test. Different letters in panels **(B, C)** indicate significant differences among conditions. **(A)** DAB staining of leaf sheath cells at the indicated times after inoculation with fungal spores. Bars correspond to 15 µm. Representative results from three independent experiments are shown. **(B)** *OsRBOHB* and *OsRBOHA* expression in leaf sheaths of rice plants grown under high Pi or Low Pi conditions, mock-inoculated and *M. oryzae*-inoculated plants. The level of *OsRBOH* transcripts was determined by RT-qPCR. Expression values were normalized to the rice *ubiquitin1* gene (*OsUbi1*). Four biological replicates (with at least 3 plants per replicate) were examined. Bars represent mean ± SEM. **(C)** MDA content in leaf sheaths of Low-Pi and High-Pi plants, mock-inoculated and *M. oryzae*-inoculated plants (left panel). Number of dead cells in *M. oryzae*-infected leaf sheaths (right panel). Asterisks indicate statistically significant differences calculated by Student’s *t*-test (**, P < 0.01; ***, P < 0.001).

DAB staining was used for cytological detection of ROS in leaf sheaths of High-Pi and Low-Pi rice plants after inoculation with *M. oryzae* spores. During biotrophic growth of *M. oryzae*, ROS strongly accumulated at the sites of pathogen entry in rice sheaths of Low-Pi plants (**Figure 4A**, left panels). Contrary to this, reduced ROS accumulation occurred in *M. oryzae*-inoculated leaf sheaths from High Pi plants (**Figure 4A**, right panels). The observed local accumulation of ROS *M. oryzae*-infected sheaths of Low-Pi plants closely resembles the HR which is generally associated with resistance to pathogen infection.

A major site of ROS production during plant/pathogen interactions is the apoplast, a process in which Respiratory Burst Oxidase Homologue (RBOH) genes are known to play a crucial role (Torres et al., 2002; Kadota et al., 2015; Qi et al., 2017; Wang et al., 2020). RBOH-mediated generation of apoplastic ROS is also an important mechanism in rice immunity impacting both the host and pathogen (Liu and Zhang, 2022). Both *OsRBOHA* and *OsRBOHB* appear to be required for the generation of ROS in rice plants under stress (Mittler et al., 2004). In particular, the involvement of *OsRBOHB* in the production of apoplastic ROS during infection by the rice blast fungus is well documented, its expression being strongly induced during incompatible rice–*M. oryzae* interactions (Takahashi et al., 2012; Chen et al., 2020). Knowing that, upon *M. oryzae* infection, ROS differentially accumulated in leaf sheaths of Low-Pi plants with respect to High-Pi plants (**Figure 4A**), we examined *OsRBOH* expression in these tissues. In concordance with results obtained by histochemical detection of ROS, a weaker induction of *OsRBOH* expression, both *OsRBOHB* and *OsRBOHA*, occurs in response to *M. oryza*e infection in leaf sheaths of High-Pi plants compared with Low-Pi plants (**Figure 4B**).

In addition to restrict pathogen progression, elevated levels of ROS might also cause damage to host cells through oxidation of biomolecules, including membrane lipids. In this work, we measured lipid peroxidation in leaf sheaths as malondialdehyde (MDA) content. MDA is a typical breakdown product of peroxidized fatty acids in plant membranes. Under no infection conditions, there were no differences in MDA content between Low and High-Pi plants (**Figure 4C**, left panel). Upon pathogen challenge, however, MDA accumulated at a higher level in Low-Pi plants than in High-Pi plants (**Figure 4C**, left panel), which correlated well with higher ROS accumulation in sheaths of Low-Pi plants (DAB staining, shown in **Figure 4A**). ROS and MDA accumulation in *M. oryzae*-infected leaf sheaths of Low-Pi plants was accompanied by a cell death reaction which was significantly reduced during the early stages of infection in High-Pi plants (**Figure 4C**, right panel).

From these results, it can be concluded that Pi accumulation in rice leaf sheaths hampers pathogen-inducible ROS production. Conversely, growing rice under a low-Pi regime favors accumulation of ROS at the infection sites early during the infection process, a mechanism used by plants to prevent the spread of infection by pathogen (or HR response).

### 3.5 Phosphate stimulates the *in vitro* growth of *M. oryzae*

**Figure 5:**
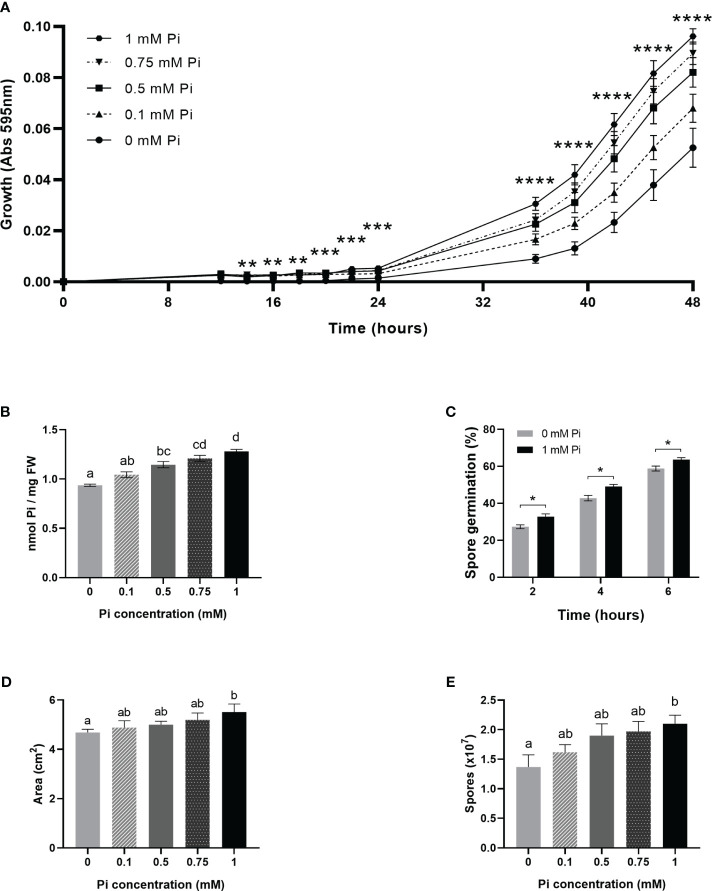
Stimulation of the *in vitro* growth and spore production of *M. oryzae* by Pi. At least three independent experiments were carried out with similar results. Asterisks in **(A, C)** denote statistical differences by Student’s *t*-test (**, P < 0.01; ***, P < 0.001; ****, < 0.0001). Different letters in **(B, D, E)** indicate significant differences among Pi conditions (one-way ANOVA). **(A)** Growth of *M. oryzae* (96-well microtiter plates with CM medium, at 25°C, in the presence of increasing concentrations of Pi: 0.1 mM Pi, 0.5 mM Pi, 0.75 mM Pi and 1 mM Pi (left panel). In control cultures, Pi was not added to the medium (0 mM Pi). Absorbances (595 nm) of fungal cultures were monitored over time. Values represent the mean ± SEM of 8 biological replicates. Statistical differences correspond to 0 mM Pi *vs* 1 mM Pi, each time point. **(B)** Pi content in fungal mycelium obtained from liquid fungal cultures supplied with Pi. Bars represent the mean ± SEM of 5 biological replicates. **(C)** Effect of Pi on the *in vitro* germination of *M. oryzae* spores. *M. oryzae* spores were germinated on CM medium (0, no Pi added; 1 mM Pi). Bar represents mean ± SEM of 3 biological replicates (N = 100 each replicate).Statistically significant differences were determined by Student’s *t*-test (*, P < 0.05). **(D)** Mycelial growth of *M. oryzae* on solid medium (CMA) containing 1 mM Pi (0, no Pi added) at 25^a^C for 5 days. Fungal growth was determined by measuring the area of mycelial growth. Data represent means ± SEM of 5 biological replicates. **(E)** Number of spores produced on solid medium (CMA) containing different Pi content (from 0 to 1 mM) after 14 days of growth. Data represent means ± SEM of 5 biological replicates.

To further establish a relationship between Pi availability and fungal growth, we evaluated the effect of Pi on the *in vitro* growth of *M. oryzae*. Towards this end, *M. oryzae* was grown in Complete Media (CM) with no Pi added to the medium (control cultures), or in the presence of increasing concentrations of Pi (0.1 mM, 0.5 mM, 0.75 mM and 1 mM Pi). Fungal growth was recorded by measuring absorbance of fungal cultures (595 nm) with time. Dose-response growth curves showed stimulation of *M. oryzae* growth when increasing the Pi concentration in the medium (**Figure 5A**). In the presence of 1 mM Pi, fungal growth was 1.83-fold higher than in control cultures (no Pi added). Determination of Pi content in fungal mycelia, confirmed that the fungus accumulated more Pi when Pi concentration increased in the medium (**Figure 5B**). The presence of Pi also had a stimulatory effect on *in vitro* germination of *M. oryzae* spores (**Figure 5C**). Equally, when grown in solid media (Complete Media Agar, CMA) supplemented with 1 mM Pi, *M. oryzae* presented higher mycelial growth and spore production than control cultures with no Pi added to the medium (**Figure 5D** and **Figure 5E**, respectively).

**Figure 6:**
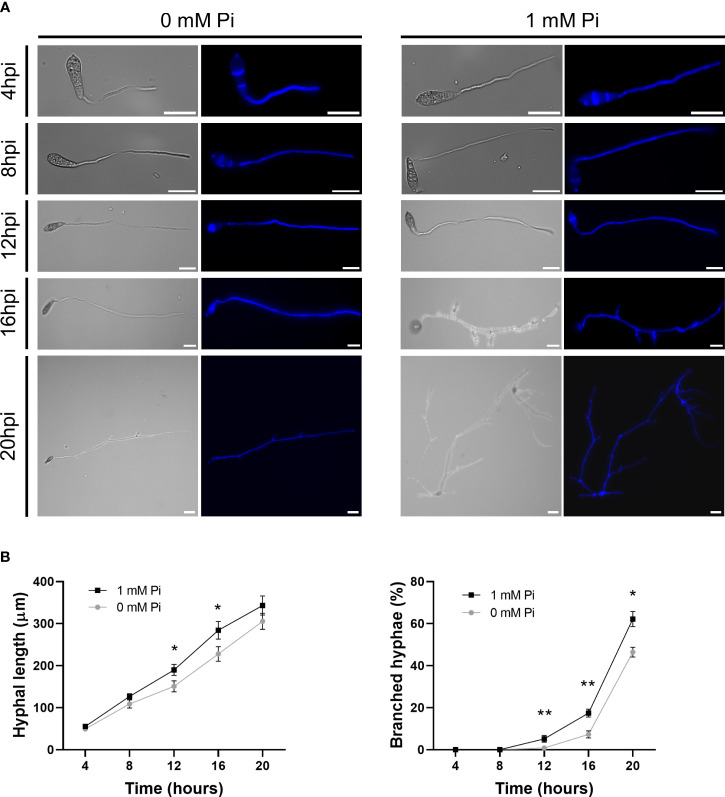
Growth and morphology of *M. oryzae* hyphae grown in the presence of Pi (1 mM Pi). No Pi was added to control cultures (0 mM). **(A)** Micrographs of *M. oryzae* cultures at the indicated times of growth in CM media. Bright field and calcofluor white staining of fungal hyphae are shown. Scale bars represent 30µm. **(B)** Hyphal length (left panel) and branching (right panel) of *M. oryzae* grown in liquid medium. Bars represent mean ± SEM of 3 biological replicates (N = 100 each replicate). Asterisks denote statistical differences by Student’s *t*-test (*, P < 0.05; **, P < 0.01).

A more detailed analysis on the effect of Pi on hyphal growth was performed by Calcofluor White staining of fungal cultures followed by microscopic observations. Calcofluor White binds components of the fungal cell walls, mainly chitin and cellulose, and allows visualization of *M. oryzae* hyphae growing in liquid media. Representative images of *M. oryzae* hyphae growing in media supplemented with Pi (1 mM), or not supplemented with Pi (0 mM Pi) at different times of growth (4 h, 8 h, 12 h, 16 h and 20 h) are presented in **Figure 6A**. In the presence of Pi (1 mM Pi), *M. oryzae* hyphae were slightly larger with more branching than hyphae in control cultures (no Pi added). Quantification of hyphal length and branched hyphae confirmed the stimulatory effect of Pi on *M. oryzae* growth and hyphal branching (**Figure 6B**).

### 3.6 Expression of *M. oryzae* phosphate transporters in Pi-treated rice plants

In addition to the deployment of pathogenic strategies, successful pathogens need to exploit nutrient supplies from their host to support their growth. Compared with production of effector, however, nutrient acquisition by phytopathogenic fungi is a less-studied aspect in plant pathogen-interactions. As the nutrient environment varies with the pathogenic lifestyle and plant growth conditions, the expression of nutrient-related fungal genes is expected to vary accordingly. Notably, the ability to acquire essential nutrients during infection is a well established virulence trait in human fungal pathogens (Ikeh et al., 2016). Whether alterations in the Pi acquisition program of plant pathogens have an impact on virulence remains elusive.

In this work, we investigated whether Pi content in the host tissue influences the *in planta* expression of *M. oryzae* Pi transporters. Initially, we mined the *M. oryzae* genome for genes encoding Pi transporters which were identified through domain and protein similarity searches as described in Materials and Methods. Proteins encoded by these genes were then classified according to the Transporter Classification Database (https://www.tcdb.org). This analysis identified genes belonging to conserved membrane transporter families that can function in Pi uptake, transport, and/or compartmentalization in *M. oryzae*. Specifically, we identified Pi transporter genes that classified in different groups of Pi transporters: i) Major Facilitator Superfamily (MFS; Group 2.A.1) which included High affinity and Low affinity Pi transporters; ii) Inorganic Phosphate Transporter PiT Family (Group 2.A.20); iii) Mitochondrial Carrier (MC) Family (Group 2.A.29); iv) Divalent Anion : Na^+^ symporter (DASS) family (2.A.47); and v) Phosphate permease family (2.A.94) (**Supplementary Table 2**).

**Figure 7:**
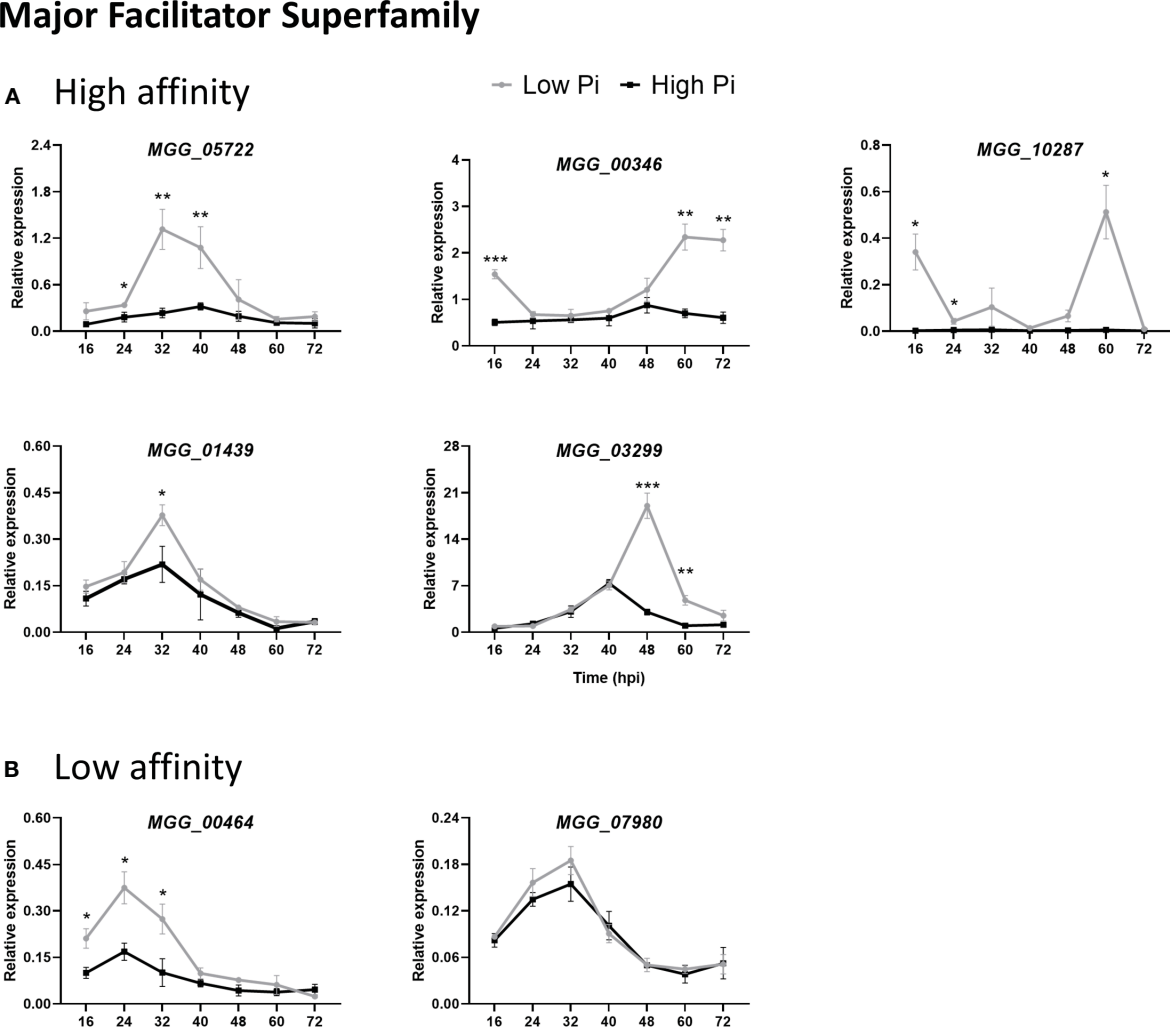
Expression of *M. oryzae* Pi transporters in the Major Facilitator Superfamily (MFS) in High-Pi and Low-Pi rice (*O. sativa* cv Maratelli) plants at the indicated times after inoculation with *M. oryzae* spores determined by RT-qPCR. The expression values were normalized *M. oryzae* actin gene (MGG_03982). Similar results were obtained in the rice cultivar TN67. **(A)** High affinity Pi transporters (MGG_05722, MGG_00346, MGG_10287, MGG_01439, MGG_03299). **(B)** Low-affinity Pi transporters (MGG_00464, MGG_07980). Data represent mean ± SEM of 4 biological replicates, each one from a pool of three different plants. Asterisks indicate significance differences as determined by Student’s *t*-test (*, P < 0.05; **, P < 0.01; ***, P < 0.001).

**Figure 8:**
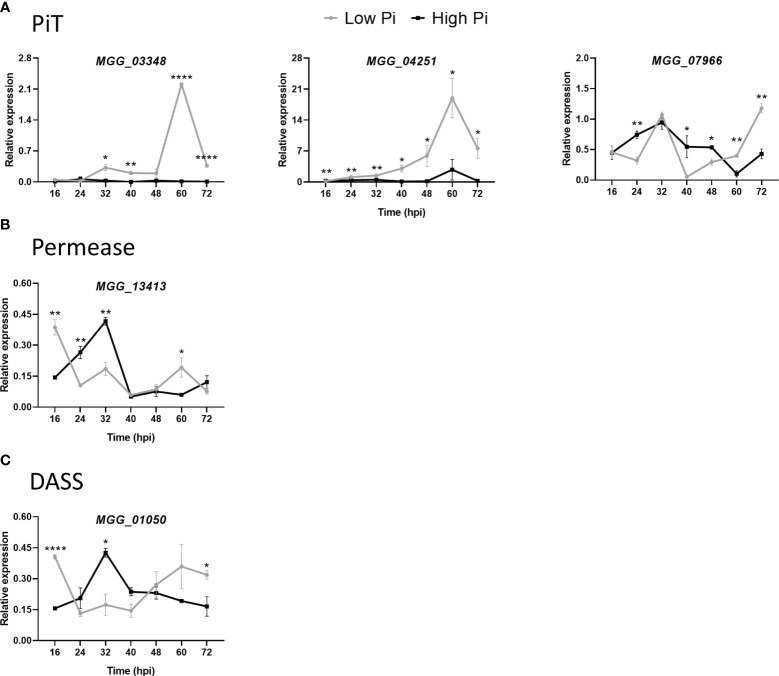
Expression of *M. oryzae* Pi transporters in High-Pi and Low-Pi rice (*O. sativa* cv Maratelli) plants at the indicated times after inoculation with *M. oryzae* spores determined by RT-qPCR. Similar results were obtained in the rice cultivar TN67. **(A)** Inorganic Pi transporter (PiT) family (MGG_03348, MGG_04251 and MGG_07966). **(B)** Pi transporter in the Pi permease family (MGG_13413). **(C)** Pi transporter in the DASS family (MGG_01050). Data represent mean ± SEM of 4 biological replicates, each one from a pool of three different plants. Asterisks indicate significance differences as determined by Student’s *t*-test (*, P < 0.05; **, P < 0.01; ****, P < 0.0001).

We measured the *in planta* expression of *M. oryzae* transporter genes in sheaths of High-Pi and Low-Pi rice plants that have been inoculated with *M. oryzae* spores, or not. The level of expression of Pi transporter genes appeared to be somewhat variable over time (16 to 72 hpi). Remarkably, the expression of high-affinity Pi uptake transporters in the MFS superfamily was strongly up-regulated in Low-Pi plants compared with High-Pi plants at one or another time point after infection (**Figure 7A**, MGG_05722, MGG_00346, MGG_10287, MGG_01439 and MGG_03299). Also, two low-affinity Pi uptake transporters in the MFS superfamily (MGG_00464 and MGG_07980) exhibited higher expression in Low-Pi plants (**Figure 7B**). The expression of Pi transporters in the PiT family (encoding Pi : Na^+^ symporters) was found to be up-regulated in Low-Pi plants, mainly during necrotrophic growth of *M. oryzae* (MGG_03348, MGG_04251 and MGG_07966) (**Figure 8A**). The Pi transporter identified in the Permease family (MGG_13413) was also up-regulated or down-regulated in High-Pi plants relative to Low-Pi plants depending on the time of infection (**Figure 8B**). Finally, a transporter belonging to the DASS family (MGG_01050) showed stronger or weaker expression in Low-Pi plants depending on the time of infection (**Figure 8C**). Although to a lesser extent, the expression of fungal mitochondrial Pi carriers also varied during infection depending on the host Pi status (**Supplementary Figure 4**).

Overall, these results are consistent with a model in which changes in Pi content in the host tissues modulate the expression of fungal genes with a function in Pi acquisition and/or homeostasis, thus, supporting growth and survival in the host tissue (e.g., by optimizing Pi acquisition from the host tissue in Low-Pi plants while avoiding potential Pi toxicity to the fungal cell in High-Pi plants). Pi-mediated alterations in the expression of *M. oryzae* Pi transporter genes might reflect mechanisms of *M. oryzae* adaptation to host Pi conditions which would then explain differences in colonization by the rice blast fungus in leaf sheaths of rice plants grown under different Pi regimes.

## 4 Discussion

In this study, we provided insight into the mechanisms underlying Pi-induced susceptibility to rice blast. We show that treatment of rice plants with high Pi, and subsequent accumulation of Pi in rice leaf sheaths, promotes host tissue invasion by *M. oryzae*. Supporting this conclusion, live-cell imaging using a *M. oryzae* isolate expressing two fluorescently-tagged *M. oryzae* effectors revealed higher rates of host cell invasion in High-Pi rice plants compared to Low-Pi plants, which was further confirmed by fungal biomass quantification. Pi accumulation in leaf sheaths also fosters the expression of effector genes encoding apoplastic and cytoplasmic effectors, these genes being essential for fungal pathogenicity. Additionally, plant cell death-inducing effectors are expressed at a higher level in High-Pi plants compared with Low-Pi plants during necrotrophy, which would then facilitate necrotrophic fungal growth in High-Pi plants. By the same time, suppressors of plant cell death are repressed in High-Pi plants. From these results, it can be concluded that Pi accumulation in rice leaf sheaths fosters the expression of fungal effectors typically associated to biotrophic colonization, as well as effectors that are produced during necrotrophic invasion (e.g., *MoCDIP* genes). Together, these results support that Pi is an important factor governing *M. oryzae* pathogenicity. Pi was also found to exert a stimulatory effect on the *in vitro* growth of *M. oryzae*.

Among the cytoplasmic effectors up-regulated in High-Pi during biotrophic invasion we identified *MoAvr-Pita*. Consistent with the observed reduction on H_2_O_2_ accumulation in High-Pi plants during infection (DAB staining), it was recently described that Avr-Pita inhibits ROS accumulation in rice (Han et al., 2021). Oppositely, a lower expression of *MoAvr-Pita* early during infection will allow ROS accumulation in Low-Pi plants. Regarding *MoBAS1*, its overexpression in *M. oryzae* was reported to promote virulence of the blast fungus (Yang et al., 2017). It is then tempting to hypothesize that up-regulation of *MoBAS1* might allow the fungus to better proliferate in High-Pi plants.

Among apoplastic effectors, *MoSLP1* was found to be strongly up-regulated in High-Pi plants early during infection (16 hpi). It is well known that this effector is capable of sequestering chitin to prevent PTI responses, such as ROS accumulation and defense gene expression (Mentlak et al., 2012). Consistent with a function of SLP1 in suppressing PTI, a more effective suppression of chitin-triggered immunity is expected to occur in High-Pi plants thereby facilitating rapid spread of the pathogen.

An interesting observation in this study was that *MoPMK1* expression was higher in High-Pi plants compared with Low-Pi plants. *MoPMK1* encodes a MAP kinase with multiple functions in *M. oryzae* growth and development, as well as in pathogenesis. In addition to appressorium development, PMK1 controls cell-to-cell movement of invasive hyphae via plasmodesmata and expression of effector genes (Sakulkoo et al., 2018; Eseola et al., 2021). PMK1 also functions as a regulator of fungal growth by controlling a series of transcriptional regulators in phosphorylation processes (Osés-Ruiz et al., 2021), thus, establishing connections between protein phosphorylation signaling and fungal pathogenicity. Knowing the multiple functions of PMK1, alterations in *MoPMK1* expression are then expected to have a strong impact on blast severity. Increased expression of *MoPMK1* in High-Pi plants might well contribute to the observed increase in *M. oryzae* virulence.

From results here presented, transition from biotrophy to necrotrophy appears to occur earlier in High-Pi plants than in Low-Pi plants. It is then reasonable to hypothesize that *M. oryzae* evolved mechanisms to sense host Pi availability and to adapt its pathogenicity program to the nutrient status in the host plant. Supporting this possibility, versatility in switching from biotrophic necrotrophic lifestyle has been proposed to occur in hemibiotrophic pathogens to meet the need for a more efficient mode of nutrient acquisition (Kabbage et al., 2015). However, to better understand the mechanisms by which Pi availability modulates lifestyle transition in *M. oryzae*, a better knowledge on factors controlling this transition is a requisite.

It is generally assumed that the time and intensity in the expression of genes encoding suppressors and inducers of plant cell death needs to be tightly controlled for successful infection by hemibiotrophic pathogens. Supporting the notion of a coordinated regulation in the expression of *M. oryzae* effectors involved in the control of plant cell death, opposite expression patterns have been observed for suppressor of cell death effectors and cell death-inducing effectors. Of interest, results here presented demonstrated that Pi content in rice leaf sheaths influences the expression of fungal genes controlling host cell death. Thus, up-regulation of cell death-inducing effectors (e.g., *MoCDIP6*, *MoCDIP7*) occurs at the late biotrophy-early necrotrophy stage in High-Pi plants, while suppressors of cell death (e.g., *MoSPD2*, *MoSPD6*, *MoSPD8*, *MoSPD3*) are weakly expressed during necrotrophy.

On the other hand, connections between nutrient sensing and pathogen virulence have long been recognized in fungal diseases of humans. In particular, Pi acquisition was found to be critical for virulence of the fungal pathogen *Cryptococcus neoformans* causing cryptococcosis in humans (Kretschmer et al., 2014). Results here presented in the rice/*M. oryzae* interaction demonstrated that Pi accumulation in the host tissue dictates the expression of fungal Pi transporter genes. Thus, up-regulation of an important number of *M. oryzae* Pi transporter genes appears to occur in Low-Pi environments within the host. Therefore, it can be hypothesized that the fungus is capable of adapting to host Pi levels, a process that might also lead to metabolic adjustments during the pathogen’s life cycle. While ensuring a more efficient mode of Pi acquisition from the host, the observed alterations in the expression of fungal Pi transporters might also contribute to avoid potential toxic effects of Pi excess. Clearly, Pi acquisition from the host tissue, and mobilization of Pi between compartments, are important factors in maintaining Pi homeostasis in the fungal cell which would have links to fungal metabolic processes contributing to fungal growth and potentially also to fungal pathogenicity (Bhalla et al., 2022). In line with this, evidence exits on the integration of Pi homeostasis with carbon and nitrogen metabolism in *M. oryzae*, as well as with the ability of the fungus to cause disease (Wilson and Talbot, 2009; Bhalla et al., 2022). As we show, increasing Pi concentration in the fungal culture medium contributes to a better mycelial growth and sporulation in *M. oryzae*. In addition to fungal growth stimulation, Pi can be considered an important factor affecting fungal pathogenicity, as demonstrated by regulation of *M. oryzae* effector genes. In other studies, ABC1 and ABC4, members of the ATP-binding cassette (ABC) superfamily of membrane transporters, have been implicated in pathogenicity of *M. oryzae* (Urban et al., 1999; Gupta and Chattoo, 2008). These findings open the way to further studies to characterize mechanisms involved in adaptation to changes in Pi availability in *M. oryzae* and to investigate whether Pi-mediated regulation of fungal Pi transporters is associated with *M. oryzae* pathogenicity.

From the perspective of the host plant, we show that treatment with high Pi was accompanied by suppression of host defense responses during *M. oryzae* infection of rice leaf sheaths. Similar results were previously reported during infection of leaves in rice plants subjected to high Pi fertilization, these plants exhibiting enhanced susceptibility to blast (Campos-Soriano et al., 2020). Early after inoculation with *M. oryzae* spores, ROS was found to accumulate, and at high levels, at the infection sites only in Low-Pi plants, but not in High-Pi plants. This is consistent with the observed up-regulation of genes encoding plasma membrane NADPH oxidases (also known as respiratory burst oxidase homologs, or RBOHs) in Low-Pi plants, these genes being the key producers of ROS in plants. Indeed, ROS production is a common mechanisms used by plants to prevent pathogen infection, and the temporal and/or spatial control of ROS production is crucial in determining the outcome of plant/pathogen interactions (e.g., resistance or susceptibility). Generally, a rapid ROS burst occurs in incompatible plant-pathogen interactions which then triggers a strong and strictly localized cell death that prevents growth of invasive hyphae. Conversely, weak and/or delayed ROS accumulation in compatible interactions usually results in susceptibility. To note, ROS were found to accumulate at the infection sites in rice leaf sheaths during incompatible interactions with *M. oryzae*, but not in compatible interactions (Chen et al., 2020). The observed strictly localized accumulation of ROS at the infection sites in Low-Pi plants is then reminiscent to the already reported accumulation of ROS during infection with avirulent *M. oryzae* (Chen et al., 2020). Oppositely, the observed reduction in ROS production in High-Pi plants correlates well with a higher rate of invasion by *M. oryzae* in these plants.

**Figure 9:**
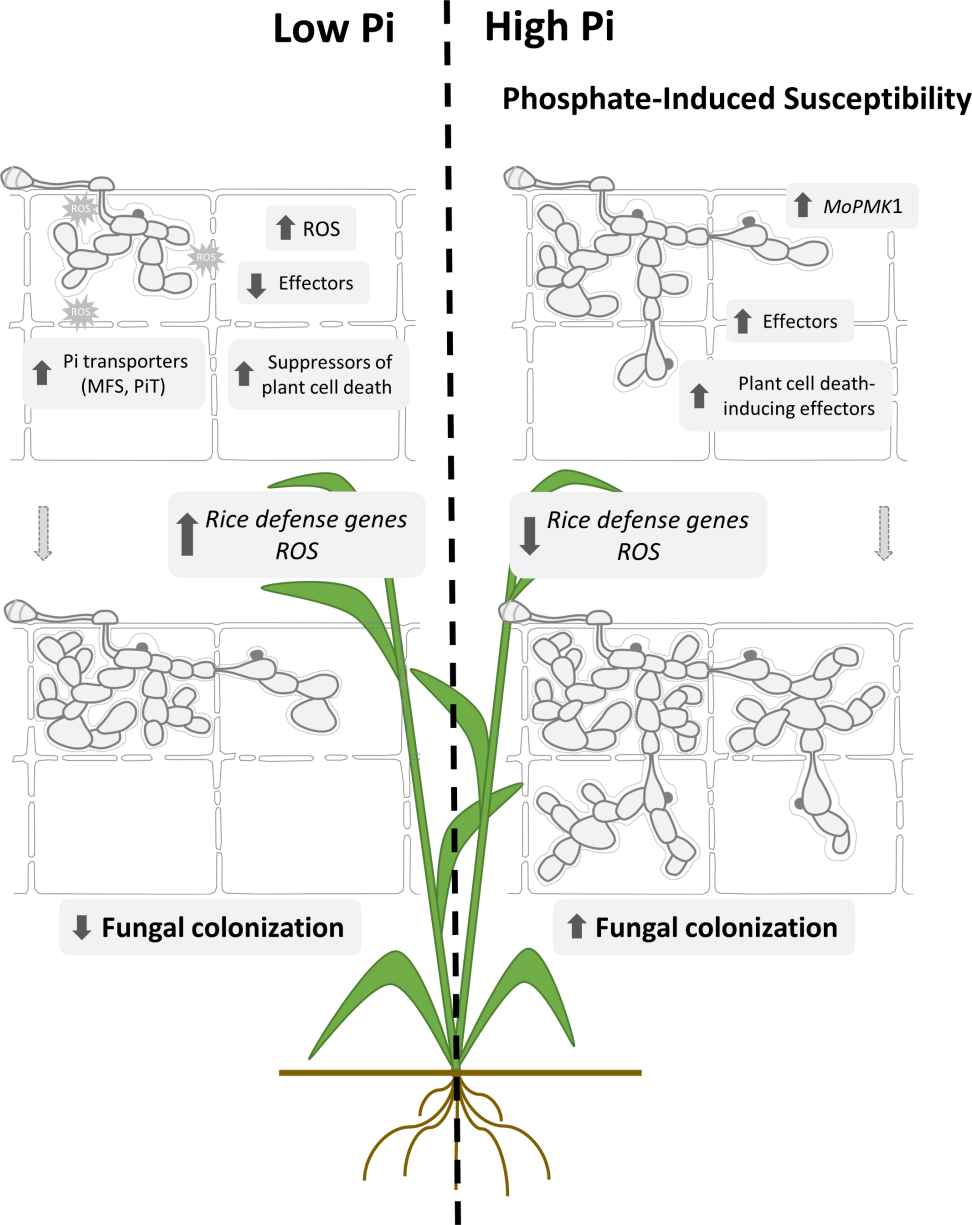
Proposed model to explain mechanisms involved in Pi-induced susceptibility to rice blast. Pi accumulation in rice leaf sheaths. Treatment of rice plants with high-Pi entails Pi overaccumulation in rice leaf sheaths. Upon pathogen challenge, stronger expression of biotrophy-associated *M. oryzae* effectors, both cytoplasmic and apoplastic effectors occurs in leaf sheaths accumulating Pi. Equally, *MoPMK1* and Plant cell death-inducing effector genes are expressed at higher levels in High-Pi plants compared to Low-Pi plants, while Suppressors of plant cell death are up-regulated in Low-Pi plants. Pi supply to rice plants causes an alteration in the expression pattern of *M. oryzae* Pi transporter genes (e.g., up-regulation of MFS and PiT family members in Low-Pi plants relative to High-Pi plants). On the plant side, Pi accumulation prevents ROS accumulation early during infection of leaf sheaths which correlates well with down-regulation of *OsRBOH* genes involved in ROS production. A weaker induction of defense-related genes also occurs during infection of leaf sheaths from High-Pi plants. Together, Pi-mediated stimulation of fungal pathogenicity factors and repression of defense-gene expression would facilitate host tissue invasion and colonization by *M. oryzae* in High-Pi plants.

Based on the results here presented, a model summarizing the effect of Pi accumulation in rice leaf sheaths during infection by the rice blast fungus in High-Pi and Low-Pi rice plants is presented in **Figure 9**. According to the model here proposed, Pi accumulation has an impact on the two interacting partners, the fungus and the host plant, with important consequences in blast disease. On the pathogen side, Pi accumulation governs the pathogenicity program of *M. oryzae* by fostering the expression of effector genes (e.g., through potentiation of fungal pathogenicity), as well as through modulation of fungal genes involved in Pi homeostasis (potentially contributing to a more efficient mode of Pi acquisition/use by the fungus in each Pi condition). On the plant side, Pi accumulation negatively affects immune responses by hampering ROS accumulation and compromising pathogen-inducible expression of defense genes. The combination of these factors would explain Pi-induced susceptibility to rice blast. These findings have important implications in rice protection because growing rice plants under a high Pi regime is expected to enhance blast susceptibility. Here it is worth mentioning that high rates of fertilizers are routinely used in rice cultivation to obtain maximum yields, while pesticides are used to reduce losses due to rice blast. These practices have a negative impact on the environment and human health. Excess of nitrogen fertilization has been also shown to increase susceptibility to *M. oryzae* infection in rice (Ballini et al., 2013; Huang et al., 2017). Knowing that Pi provokes an increase in fungal pathogenicity as well as a reduction in the capability of the rice plant to defend itself from pathogen attack, efforts must be taken to avoid problems associated with inadequate supply of Pi fertilizers in rice farming. A better understanding about Pi regulation of fungal pathogenicity and host immunity in the rice/*M. oryzae* interaction will prove pivotal in controlling rice blast disease. The information gained during this study on the underlying mechanisms that rice plants use to cope with Pi excess will help in developing more sustainable solutions to meet the growing demand for rice production while minimizing environmental impact.

## Data availability statement

The original contributions presented in the study are included in the article/**Supplementary Material**. Further inquiries can be directed to the corresponding author.

## Author contributions

HM-C: Data curation, Formal Analysis, Investigation, Methodology, Writing – review & editing. MB: Formal Analysis, Investigation, Writing – review & editing. BV-T: Formal Analysis, Investigation, Writing – review & editing. BS: Conceptualization, Formal Analysis, Funding acquisition, Project administration, Supervision, Writing – original draft, Writing – review & editing.
