# Supplementary material for: Phosphate accumulation in rice leaves promotes fungal pathogenicity and represses host immune responses during pathogen infection
Source: Front Plant Sci. 2024 Jan 17;14:1330349. doi: 10.3389/fpls.2023.1330349 (PMC10827867; doi:10.3389/fpls.2023.1330349)
Supplement: Supplementary file 1 [file DataSheet_1.docx]

Supplementary Material

Phosphate accumulation in rice leaves promotes fungal pathogenicity and represses host immune responses during pathogen infection

Héctor Martín-Cardoso^1^, Mireia Bundó^1^, Beatriz Val-Torregrosa^1^, Blanca San Segundo^1, 2 *^

^1^ Centre for Research in Agricultural Genomics (CRAG) CSIC-IRTA-UAB-UB, C/ de la Vall Moronta, CRAG Building, Campus Universitat Autònoma de Barcelona (UAB), Bellaterra (Cerdanyola del Vallés), Barcelona, 08193, Spain.

^2^ Consejo Superior de Investigaciones Científicas (CSIC), Barcelona, Spain.

* Corresponding Author, Blanca San Segundo,

E-mail: [blanca.sansegundo@cragenomica.es](mailto:blanca.sansegundo@cragenomica.es); blanca.sansegundo@csic.es

**A**

**B**


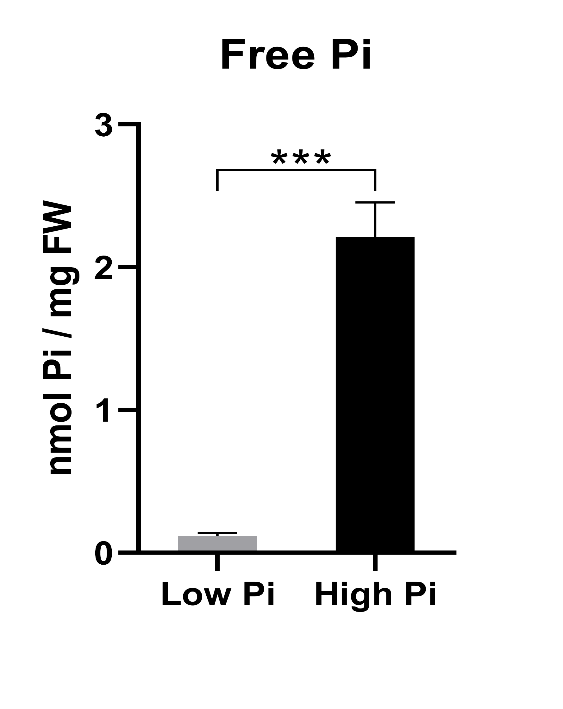


7 days

14 days

***M. oryzae infection***

**Water**

**Low Pi**

**High Pi**

7 days

14 days

**SUPPLEMENTARY FIGURE 1**

Pi treatment of rice plants. (**A**) Experimental design used for Pi treatment and infection of leaf sheaths with *M. oryzae*. Rice plants were watered for 7 days and allowed to continue growth under low Pi or high Pi for 14 days (0.025 mM Pi and 2.5 mM Pi, respectively). Rice leaf sheaths were excised from Low-Pi and High-Pi plants and inoculated with a suspension of *M. oryzae* spores (Guy11; 5 x 10^4^ spores/ml). (**B)** Free Pi content in leaf sheaths of rice plants that have been grown under low or high Pi as indicated in A. Bars represent mean ± SEM of 4 biological replicates with three plants each replicate. Asterisks indicate significance differences as determined by Student’s *t*-test (***, P ˂ 0.001).

Low Pi

High Pi


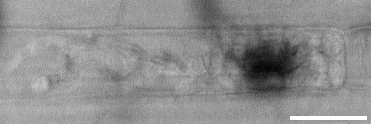

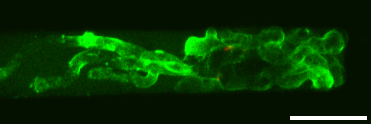

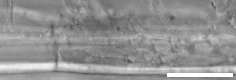

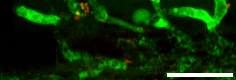


BIC

BIC

BIC

BH

BH

BH

BH

Ap

**SUPPLEMENTARY FIGURE 2**

Confocal microscopy during biotrophic invasion of rice cells at 32 hpi. GFP fluorescence corresponds to the apoplastic effector BAS4 accumulating at the EIHM compartment. Red fluorescence corresponds to the cytoplasmic effector PWL2 accumulating at the BICs. Scale bars represent 20 µm. Ap, Appressorium; BIC, Biotrophic Interfacial Complex; BH, Bulbous Hyphae; PH, Primary Hyphae. Arrowheads indicate cell to cell of *M. oryzae*.

**SUPPLEMENTARY FIGURE 3**

Number of appressoria formed in *M. oryzae*-inoculated sheaths of High-Pi and Low-Pi plants. Visualization of *M. oryzae* appressoria in leaf sheath segments (1 cm in length) was carried out at 8 hpi. Error bars indicate the first and third quartiles (n = 15). The horizontal line within the box represents the median value (i.e., 50^th^ percentile). Three independent experiments were carried out with similar results.

**SUPPLEMENTARY FIGURE 4**

Expression of *M. oryzae* Pi transporters in the Mitochondrial Carrier (MC) family (MGG_02370, MGG_07489, MGG_13428 and MGG_09906) in High-Pi and Low-Pi rice plants at the indicated times after inoculation with *M. oryzae* spores. Expression was determined by RT-qPCR. The expression values were normalized to the *M. oryzae* actin gene (MGG_03982). Data represent mean ± SEM of 4 biological replicates, each one from a pool of three different plants. Asterisks indicate significance differences as determined by Student’s *t*-test (*, P < 0.05; **, P < 0.01; ***, P < 0.001).
