# Supplementary material for: Phosphate accumulation in rice leaves promotes fungal pathogenicity and represses host immune responses during pathogen infection
Source: Front Plant Sci. 2024 Jan 17;14:1330349. doi: 10.3389/fpls.2023.1330349 (PMC10827867; doi:10.3389/fpls.2023.1330349)
Supplement: Supplementary file 2 [file Table_1.docx]

**SUPPLEMENTARY TABLE 1.**  List of oligonucleotides used in this study.

| **Gene name** | **Gene ID** | **Sequence 5'->3'** | |
| --- | --- | --- | --- |
| **For sequencing** | | | |
| BAS4 | MGG_10914 | CCGACTCGCACCAGAATCTT | |
| eGFP |  | CGACAACCACTACCTGAGCA | |
| PWL2 promoter |  | TATGGTCCCGGGTGATAAAA | |
| PWL2 | MGG_13863// MGG_04301 | CAGGCACGGATATCATGTTG | |
| mCherry |  | GACCACCTACAAGGCCAAGA | |
| **For RT-qPCR** | | | |
| *OsUBQ1* | Os06g0681400 | F | TTCCCCAATGGAGCTATGGTT |
|  |  | R | AAACGGGACACGACCAAGG |
| *Mo28*S |  | F | TACGAGAGGAACCGCTCATTCAGATAATTA |
|  |  | R | TCAGCAGATCGTAACGATAAAGCTACTC |
| *OsPBZ1* | Os12g0555500 | F | GCGTTTGAGTCCGTGAGAGT |
|  |  | R | GCAGAACACATTCAGACTTGCC |
| *OsPR1a* | Os07g0129200 | F | CTGCAAGCTGGAGCACTCG |
|  |  | R | AAGATGTTCTCGCCGTACTTCC |
| *MoActin* | MGG_03982 | F | TTCATGTTACTTTCGCGGCC |
|  |  | R | CTAGCGACTCCGGTAAAGCG |
| *MoPWL2* | MGG_13863// MGG_04301 | F | GACAAAGGCGAAAGAGAGGG |
|  |  | R | CCAGGATAACTGGGGCCATA |
| *MoAvr-Pita* | MGG_15370 | F | ATGGTTGCGAATGGGATGGG |
|  |  | R | AGCATAACTGTCGGGGTTTTT |
| *MoBAS1* | MGG_04795 | F | GCCGACCAAGGTTCTAACACA |
|  |  | R | TGTTTTTCCTCACGAATCGGTC |
| *MoBAS107* | MGG_10020 | F | TCAATCGCAGGAGAAGTCGC |
|  |  | R | TCGTGCCCTATCAAAGCAGA |
| *MoBAS170* | MGG_07348 | F | TTCGCCCTGTTTGTCCTCAA |
|  |  | R | CATGTCGCCGGTGGCTTTA |
| *MoBAS83* | MGG_08506 | F | AGCTGGAAGTGGAGAAAGGC |
|  |  | R | GACCGAAACGACCAAGTCCA |
| *MoBAS4* | MGG_10914 | F | CCGACTCGCACCAGAATCTT |
|  |  | R | CTGTTGTTGTCGGGGTAGGT |
| *MoSLP1* | MGG_10097 | F | CTCCCCCTACTACACCATCG |
|  |  | R | GAGGTTGAACTTTTGGGCGAC |
| *MoBAS113* | MGG_05785 | F | GCTCGTCGGCATTTTTAGCA |
|  |  | R | TGGTGAACTTGATGTCCTCGG |
| *MoPMK1* | MGG_09565 | F | CCAATCCACCAAGCAACT |
|  |  | R | TGATCTTCTTTATGGCAACC |
| *MoSPD2* | MGG_12942 | F | GACTTTTGCCAGTGCGGC |
|  |  | R | GGGGCAGAGTCCGTAAAAGG |
| *MoSPD6/MoBAS3* | MGG_11610 | F | TAACTTGGGAGCAGTGGCCTA |
|  |  | R | GCTCAAACTTCCAACCTCCCC |
| *MoSPD8/MoBAS162* | MGG_09379 | F | ACGACCAGTGTTTGAGCCAT |
|  |  | R | CGCGATATTCCGAGGTCTGG |
| *MoSPD3/MoBAS52/MoHEG13* | MGG_09378 | F | ACCCTCCTGCTTGTTTCTGTC |
|  |  | R | TTTTGCTGCCTTGGTTCCAG |
| *MoCDIP5* | MGG_10234 | F | CTGGTCGCCAACAAACAAACA |
|  |  | R | TTTTGGAGCGACCAGTTGAG |
| *MoCDIP6* | MGG_01532 | F | AGTTCATCACCCCCACTGTC |
|  |  | R | TGAAACCCCTCTGCTGCTTG |
| *MoCDIP7* | MGG_03354 | F | CAAGCCGTCCAGCACTCTC |
|  |  | R | ACCGTCGGAGGAGTAAAGGT |
|  | MGG_05722 | F | GTAGAAGGCGGGGAGAATCG |
|  |  | R | GCAAAGAGCAAGCGACCATC |
|  | MGG_10287 | F | AAGGTCAAGGGTCAACAGGC |
|  |  | R | ACCACACACAAAGGGTTCCA |
|  | MGG_00346 | F | CTTTGGTGGGCGGTTGATTG |
|  |  | R | ACCGAGATGAACTGGCCTTG |
|  | MGG_07980 | F | AGGCTGCGTTTCTGATAGGG |
|  |  | R | CGCCTCGGGAATGACAAACA |
|  | MGG_00464 | F | AGTTTGGCCTTTGCCTCTTT |
|  |  | R | GGCAGGACAGAAACCGTCTAA |
|  | MGG_01439 | F | TATTCCTTCCGCTTCGTGGC |
|  |  | R | GTCAAGAGAGAACCACGGCA |
|  | MGG_03299 | F | TTCACGACCCTTCTGCTTCC |
|  |  | R | CCTCAACCACGTCGGAAACT |
|  | MGG_13413 | F | AAGTCATTCGCCCTGCTCAA |
|  |  | R | CGGTAGGTAGGTTTGGCGTT |
|  | MGG_07966 | F | CATCCTGTCATCATCTCCCCC |
|  |  | R | ATGCCACTTCTGTGAGGACG |
|  | MGG_03348 | F | CCTGGAGGACCATCAACTGG |
|  |  | R | AACGATACCAGAGACGGGGA |
|  | MGG_04251 | F | GCACACCTACTCCTTCGTCC |
|  |  | R | GGACCGACCGAGTTTCCAAT |
|  | MGG_01050 | F | TATGCTAAAGCGCAGGGTGG |
|  |  | R | CTTGAGCACCTTGCGGAAAC |
|  | MGG_02370 | F | GCTCTTTCGGTGGTATCGGT |
|  |  | R | CCGTAGATGGCGAACTGACC |
|  | MGG_07489 | F | ACAACAAGGGCATGATCGGT |
|  |  | R | CCGAATCCCGTCAACAAAGC |
|  | MGG_13428 | F | CGCGGTTTCTACTCTGGCTT |
|  |  | R | CACCGCAAACTGACCAACTG |
|  | MGG_09906 | F | TTTTGGCTACTCGGCTCAGG |
|  |  | R | ACTGGTGAGCGTATTCTGGG |
